# Multilayer modeling and analysis of human brain networks

**DOI:** 10.1093/gigascience/gix004

**Published:** 2017-02-06

**Authors:** Manlio De Domenico

**Affiliations:** Departament d’Enginyeria Informàtica i Matemàtiques, Universitat Rovira i Virgili, Av.da Països Catalans, 26, 43004 Tarragona, Spain

**Keywords:** multilayer networks, functional connectivity, structural reducibility, versatility

## Abstract

Understanding how the human brain is structured, and how its architecture is related to function, is of paramount importance for a variety of applications, including but not limited to new ways to prevent, deal with, and cure brain diseases, such as Alzheimer’s or Parkinson’s, and psychiatric disorders, such as schizophrenia. The recent advances in structural and functional neuroimaging, together with the increasing attitude toward interdisciplinary approaches involving computer science, mathematics, and physics, are fostering interesting results from computational neuroscience that are quite often based on the analysis of complex network representation of the human brain. In recent years, this representation experienced a theoretical and computational revolution that is breaching neuroscience, allowing us to cope with the increasing complexity of the human brain across multiple scales and in multiple dimensions and to model structural and functional connectivity from new perspectives, often combined with each other. In this work, we will review the main achievements obtained from interdisciplinary research based on magnetic resonance imaging and establish *de facto*, the birth of multilayer network analysis and modeling of the human brain.

## Background

Brain networks provide a map of the complex organization, either structural or functional, of its units. In the last decades, several experimental measurements, based on electro-encephalography, magneto-encephalography (MEG), diffusion tensor imaging (DTI), structural and functional magnetic resonance imaging (fMRI), have been carried on to explore such an organization [1,2].

In this context, networks consist of brain regions (i.e., the nodes) and their structural or functional connection patterns (i.e., the edges) obtained by evaluating cross-correlation or more sophisticated similarity measures in space, time, and frequency domains.

Structural networks usually represent an anatomical parcellation of the brain where links among neurons or regions, encoding physical connections, are obtained from MRI, DTI, or histological data. In DTI, one of the widest adopted techniques, the diffusion of water molecules and the result of their interactions with tissues are measured with high accuracy, allowing the reconstruction of nerve fibers and mapping of the human brain in three dimensions with exceptional resolution. The functional connectivity of the brain is usually obtained by measuring a specific type of physical signal (e.g., blood oxygen level – dependent contrast as in fMRI or magnetic field as in MEG) from different regions and then comparing pairwise signals by means of some similarity measure (e.g., cross-correlation, transfer of entropy, spectral coherence, etc.). If the similarity between two signals is statistically significant, a functional link is considered between the corresponding brain regions. Many studies differ in the type of signal they measure and the statistical methodology adopted to build the functional network, but they all share the approach described above.

Network modeling approaches successfully unveiled interesting features such as small-worldness – where the underlying topology is highly locally clustered and the presence of long-range connections dramatically reduces the distance between units – and modular and rich-club organization – where the underlying topology can be coarse-grained and described as a network of modules with highly-connected units tending to be connected to each other more frequently than random expectation. The success of network mapping increased, in parallel, the need for novel methodologies devoted to unraveling the structure and the function of the brain at multiple spatial and temporal scales [3,4]. However, the lack of an appropriate mathematical framework for the representation and analysis of multivariate connectivity data forced many studies to neglect, disregard, or aggregate available information in order to cope with the high amount of underlying complexity.

More recently, researchers explored the possibility of studying the human brain without necessarily either throwing out or aggregating the data deluge available nowadays. An important and promising approach is the use of multilayer networks (see [5,6] for a thorough review), recently developed to provide a mathematical framework [7] to model and analyze complex data with multivariate and multi-scale information. Recent results from this research direction are exciting and provide new insights about our understanding of the structure and function of the human brain.

## Multilayer network representation of the human brain

A multilayer network consists of several distinct classical networks, each one encoding a specific type of information about the system. In the following, we will briefly discuss different types of multilayer brain networks where layers’ connectivity, measured with respect to a specific definition of similarity (e.g., cross-correlation, spectral coherence, etc.) might encode (i) activity in different frequency bands, (ii) time-varying activity, (iii) activity with respect to different tasks, and (iv) structural and functional connectivity.

**Figure 1:**
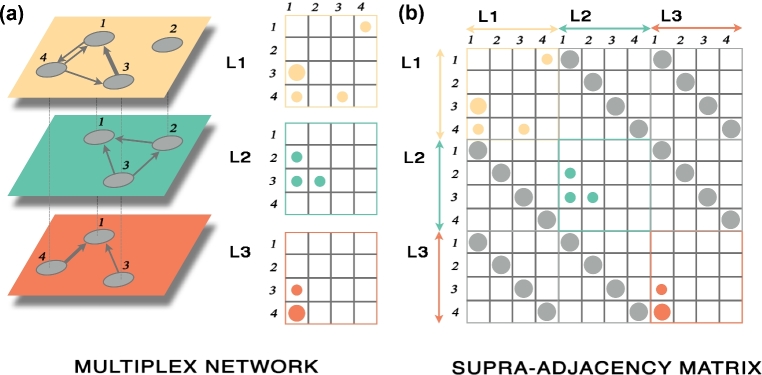
Multilayer network representation. **(a)** A multilayer network consists of different networks encoded by layers, each one represented by a (possibly directed and weighted) adjacency matrix. **(b)** The rank-4 multilayer adjacency tensor, representing intra- and inter-layer connectivity, is generally flattened by matricization to a rank-2 tensor, generally known as supra-adjacency matrix, without loss of information.

While standard networks can be represented by adjacency matrices, indicating the presence and the intensity of connections among the system’s units, multilayer networks require higher-order matrices, i.e., tensors, to be appropriately represented (see Fig. 1a) [7]. In general, the components of the multilayer adjacency tensor of *N* nodes and *L* layers are indicated by $M^{i\alpha }_{j\beta }$ and encode the connectivity between unit *i* in layer α and unit *j* in layer β, with *i, j* = 1, 2, …, *N*. For instance, intra-layer connectivity in the αth layer is given by the entries $M^{i\alpha }_{j\alpha }$. A standard approach is based on flattening this rank-4 tensor into a rank-2 tensor, named the supra-adjacency matrix, with a block structure where diagonal blocks encode intra-layer connectivity and off-diagonal blocks encode inter-layer connectivity (Fig. 1b).

The tensorial representation of multilayer networks allows us to develop a powerful mathematical framework to extend traditional complex network analysis such as detection of modular super-units [8,9] and identification of most central units [10]. The majority of such tools are based on the analysis of how information spreads through the multilayer system (see [11] and references therein) and provides a suitable framework for the structural analysis of the human brain.

While several classical network concepts have been successfully and satisfactorily extended to multilayer systems, approaches adopted to model the human brain are mainly based on multiplex and interconnected multiplex topologies. In both models, the same node is usually replicated on more than one layer, where it exhibits different connectivity patterns depending on the information encoded by the layer. A multiplex topology is an edge-colored multigraph consisting of different layers that are not interconnected to each other: $M^{i\alpha }_{j\beta }=0$ for any *i, j* = 1, 2, …, *N* and α, β = 1, 2, …, *L* (with α ≠ β), using the notation introduced before. An interconnected multiplex topology includes links across layers, although only the ones among the node’s replicas are allowed: $M^{i\alpha }_{j\beta }=0$ for any *i* ≠ *j* and α ≠ β, whereas $M^{i\alpha }_{i\beta }\ne 0$ for α ≠ β (as in Fig. 1a). Other multilayer network models are possible, but they have found a few applications in neuroscience, if any.

It is worth remarking that one should be cautious in the choice of the network model to adopt for the analysis of the human brain, if one is interested in exploiting the tensorial algebra developed to naturally extend the majority of classical network descriptors to the multilayer realm [7]. In fact, when interconnectivity is absent, analysis based on the multilayer adjacency tensor provides the same results as classical analysis of each layer separately.

In the following, we will consider applications involving interconnected multiplex networks, and we will refer to them as ‘multiplex’, for the sake of simplicity.

## Frequency-based decomposition

Frequency-based decomposition is an approach that provides a multilayer functional representation of the human brain. In the case of fMRI, signals are filtered and components between 0.01 and 0.1 Hz are usually kept [12–14] (see [15] for a review). The choice of the frequency band might have a deep impact on the functional representation of the brain. In fact, standard methodologies do not distinguish the contributions coming from different frequency bands, considering only one specific range. The resulting network provides a functional map of the brain and allows for identification of special regions that act as hubs, i.e., units either with larger connectivity than others or with strategic functions that maximize the information flow through them [16–19] (see [15] for a review). It is worth remarking that while the concept of information flow is well defined for structural networks, it requires care in the case of functional ones. In fact, functional representations encode statistically significant correlations between brain regions, and, strictly speaking, the concept of information flowing through such (often non-physical) links is poorly defined. Here, the interpretation of some network descriptors in terms of information flow is given to better elucidate the meaning of the descriptor in a structural context, rather than to characterize physical information dynamics.

Given their functional importance, hubs mediate interactions among other regions and might favor the brain’s integrated operation. They are generally identified by centrality descriptors [20], and they are of particular interest in many applications [21–24]. Recent studies have shown that the importance of each region is subjected to dramatic changes depending on the frequency cuts [25] and that hubs might be very different when functional connectivity is measured in different frequency bands [26]. These results, together with previous findings concerning the importance of topological information measured from components above 0.1 Hz [27–29], suggest that a novel framework for modeling and analysis of the human brain’s functional connectivity is required.

**Figure 2:**
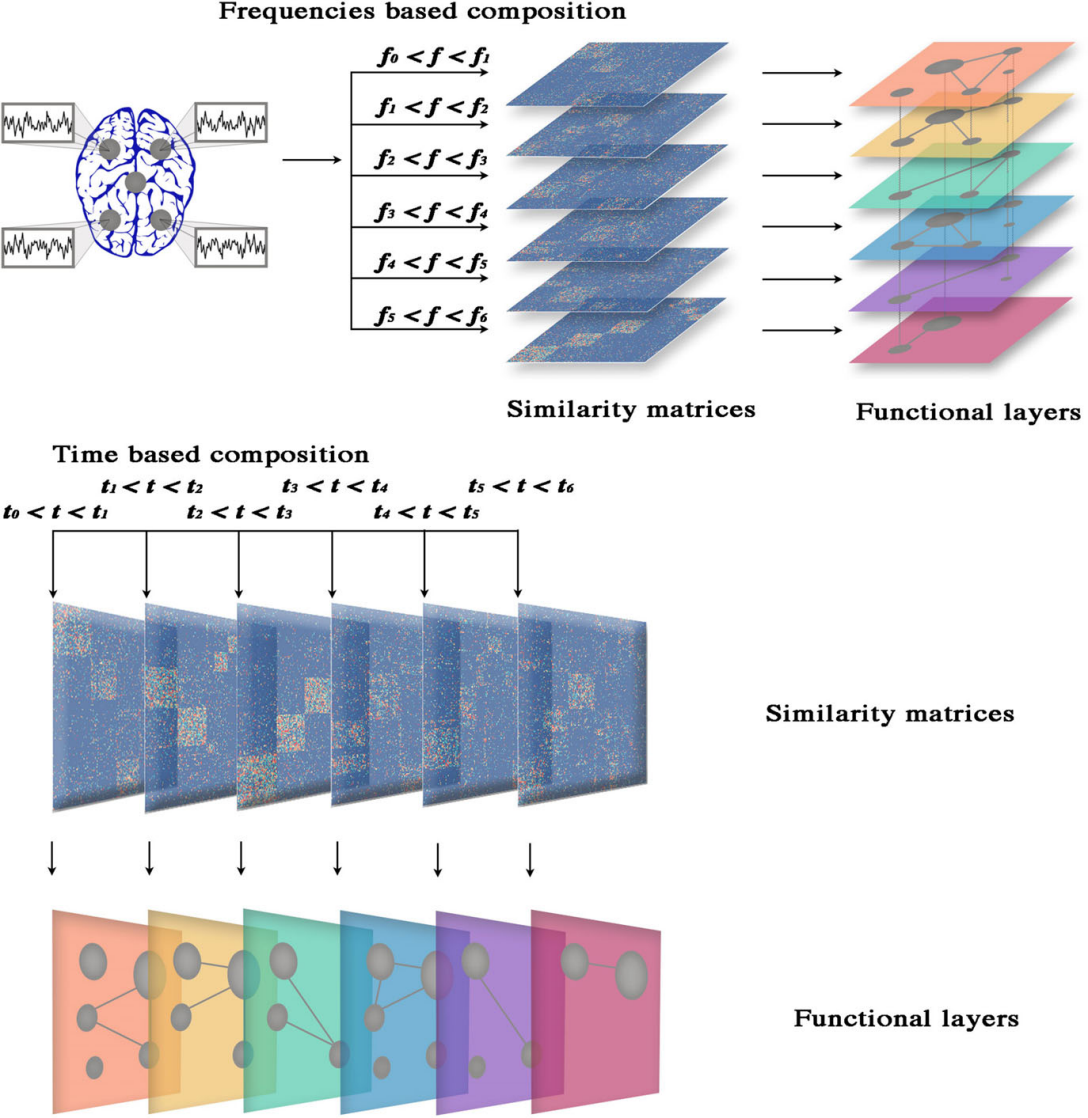
Building the multilayer functional brain. **Top panels:** brain activity is measured in different regions, and signals are decomposed in the frequency domain. The frequency domain consists of (possibly overlapping) frequency bands, and for each band, coherence – or other similarity descriptors – is measured between all pairs of regions. A similarity matrix is built for each frequency domain, and statistical analysis of significance is used to map each matrix into a functional network, constituting a functional layer of the overall multilayer system. **Bottom panels:** in this case, signals are decomposed in the time domain, which consists of (possibly overlapping) consecutive temporal snapshots. A similarity matrix is calculated for each snapshot, and the corresponding functional layer is built.

**Figure 3:**
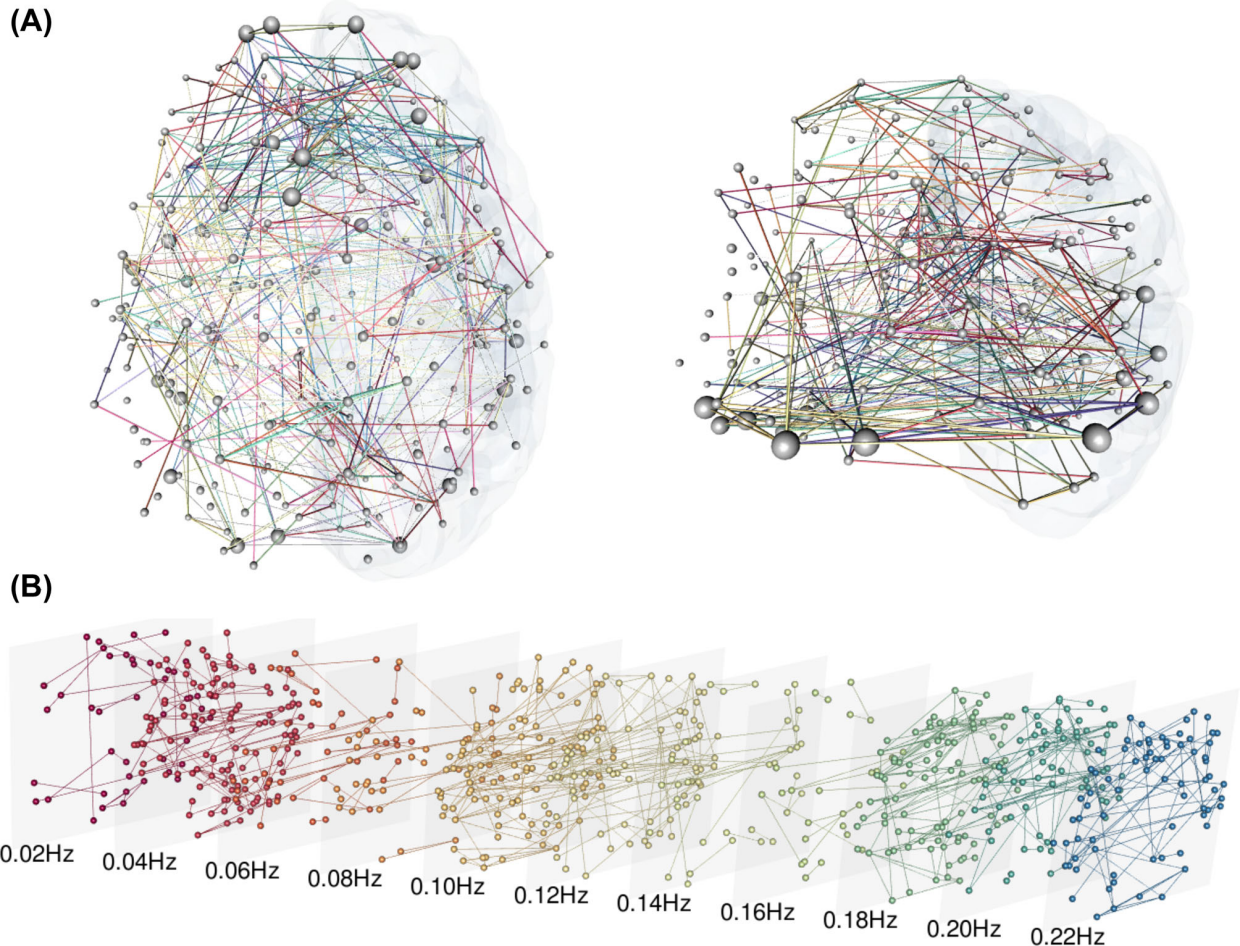
Visualizing the multilayer functional brain. Three-dimensional representations of the multilayer functional brain of a schizophrenic subject, based on frequency decomposition (11 layers, non-overlapping frequency bands between 0.01 and 0.23 Hz). Only links with at least 6 standard deviations from the mean are shown (see [30] for further details). **(a)** Edge-colored representation, where connections are colored according to the frequency band and node size is proportional to their functional versatility [30]. **(b)** Multi-slice representation, where each layer encodes information about a specific frequency band [73] and inter-layer connectivity is not shown explicitly for the sake of simplicity. The color scheme is the same in the two representations.

The new framework must be able to consider functional information from different frequency bands simultaneously: in practice, for each band it is sufficient to build a functional network and then to analyze the resulting system as a whole. Multilayer networks provide the mathematical background [7] for this purpose. In this new framework, each region of the brain is mapped into a network node and replicated across all layers, encoding frequency bands, where they are connected with other nodes by means of functional links – corresponding to significant correlations in a specific frequency band. The methodology is summarized in the top panels of Fig. 2, while the result of the procedure applied to a real human brain is visualized in Fig. 3.

Nodes are interconnected with their replicas – also known as ‘state nodes’ – across layers, and the weight of these links is, in general, a free parameter that must be estimated from the data or by maximizing a specific cost function [30]. For each unit, the set of state nodes constitutes a ‘physical node’ corresponding to a specific brain region. State nodes of a single physical node are interconnected categorically; i.e., they build a clique.

The first question to answer is to what extent such an enriched representation of functional connectivity is more valuable than other aggregated (or less rich) representations. The answer has been recently given in De Dominico et al. (2016), where it has been shown that each functional layer – in a range between 0.01 and 0.25 Hz, in steps of 0.02 Hz – provides unique information and should be neither aggregated with other layers nor neglected [30]. The result is based on the analysis of structural reducibility [31], a modern technique grounded in information entropy.

The irreducibility of the multilayer functional representation of the human brain raises the necessity for multilayer analysis of the underlying architecture, and a few first results have been recently reported about the identification of hubs. In other contexts, it has been shown that hubs in a multilayer network might be dramatically different from hubs in each layer of the system [32]. An intuitive example is given in the following. Let us consider a two-layer system where a certain node is in the periphery of both networks, and let us consider that such a node is the only one common to the two layers. It is clear that this node is crucial for the exchange of information between the two layers, and as a consequence, it will be most central with respect to this criterion. In a classical analysis, where the layers are considered separately, the node is still peripheral and it would be the less central (information exchange can be modeled by bits diffusing through the system either along random walks [33] or shortest paths [34,35] between two endpoints).

The multilayer analysis of brain regions, centrality reveals that hubs are, in general, different from the hubs identified by standard methodologies based on single-layer network analysis. The most surprising finding is that such hubs can be used to distinguish, with high accuracy and sensitivity (above 80% in both cases), the brain of a schizophrenic patient from a healthy brain in resting state [30], thus improving our understanding of schizophrenia and opening the door to the analysis of other brain disorders within the same framework.

Magnetoencephalography has been recently used in a similar spirit, with layers encoding the connectivity between neural oscillations within four frequency bands, namely alpha (8–13 Hz), beta (13–30 Hz), low gamma (30–50 Hz), and high gamma (50–100 Hz). In this context, the mean connection strength – averaged across the network where the functional connectivity between schizophrenic patients and controls differs most – has been used to gain new insights about, within, and between oscillatory frequencies [36]. Two regimes of multilayer network behavior have been identified in a system with five layers (bands 1–4, 4–8, 8–13, 13–30, and 30–48 Hz): in the first regime, layers are independent, while in the second regime they are highly dependent. Results suggest that the healthy human brain operates at the transition point between these two regimes [37].

These studies provide evidence and support for the hypothesis that functional layers do not act as independent entities, suggesting the existence of mechanisms for integration and segregation of brain activity within and across different frequency bands. Very recently, a mechanistic model for this process has been proposed [38]. The authors have compared the performance of two models: in model A, each brain region generates oscillations in a single frequency; in model B, each brain region can generate oscillations in multiple frequency bands. Model B, named the ‘multi-frequency model’, does not take into account cross-frequency interactions, but it still outperforms the single-frequency model in reproducing empirical MEG data [38].

In a more recent work, MEG recordings during resting states in subjects affected by Alzheimer’s disease have been used to build a multilayer network where layers represent functional connectivity in different frequency bands (2–4, 4–8, 8–10.5, 10.5–13, 13–20, 20–30, and 30–45 Hz). The study has provided evidence that regional connectivity in unhealthy subjects was abnormally distributed across frequency bands – a feature with no counterpart in healthy individuals – revealing an abnormal loss of inter-frequency centrality in memory-related association areas. The proposed methodology has led to high-classification accuracy (78.4%) and sensitivity (91.1%) of subjects, confirming the superior performance of multilayer analysis as compared to more traditional approaches [39].

All the results briefly described in this section support and reinforce the possibility of adopting multilayer techniques as potential non-invasive biomarkers for neurodegenerative diseases and mental disorders.

## Time-varying network model and task-based decomposition

Instead of building functional layers in the frequency domain, it might be desirable to consider the brain activity in the time domain because temporal changes and their mapping might be biologically meaningful. It is worth noting that historically this was, in fact, the first multilayer approach to the analysis of brain networks, even when a formal theory for this type of structure was not yet available [40].

Usually, the measured blood oxygen level–dependent activity is divided into a series of time windows named snapshots, which can be overlapping or not, and a pairwise measure of correlation between regions of interest is calculated to build a functional network for each snapshot. However, it is fundamental to remark that this processing phase is far from providing a rigorous and well-established method to build functional networks from this type of data [41]. In practice, overlapping and non-overlapping windows are not statistically independent [42,43], their length is a free parameter, and their choice requires careful inspection of the data [44,45] to avoid mapping spurious connectivity fluctuations.

The resulting network is a multilayer graph where each layer corresponds to a functional snapshot of brain activity. This approach has the advantage of building a static backbone of the underlying functional dynamic of the human brain that can be used, for instance, to better understand how it operates during specific tasks or at the onset of an epileptic seizure. In this regard, multilayer networks describing how functional connectivity changes across time provide a richer framework than traditional approaches [46]. In this framework, state nodes are interconnected only with their subsequent replicas, like in a chain. This methodology, summarized in the bottom panels of Fig. 2, has opened the door to several studies and triggered the development of novel theoretical measures to identify the most influent brain regions during learning [47] and how they cluster together in functional modules [48], to cite some of them.

The multilayer model for time-varying networks can be used to explore the role of functional fluctuations while in a resting state or performing specifying activities (see [3] for an up-to-date review), where in the latter case one defines a task-based representation of brain activity [48,49]. This type of decomposition is of particular interest because it is possible to map the reconfiguration of brain regions’ correlated activity between different tasks or during a learning process [50,51].

Besides the variety of its applications, very recently this novel framework has been used to better characterize high-level language processing in humans by using fMRI data from 22 human subjects asked to perform a language comprehension task. While it is known that the activity of left frontal, temporal, and parietal cortices is very correlated, constituting a functional system, when an individual is performing a natural language comprehension task or she is resting, it is still poorly understood how those brain regions become part of such an integrated functional system. By identifying functional modules within the multilayer framework, involving the generalization of classical modularity maximization to the multilayer domain [8], it has been shown that a stable core of mutually co-activating brain regions emerges mainly in the left hemisphere, whereas a periphery of brain regions is developed in the right hemisphere while co-activating with different regions at different times. One might ask if the use of such a complicated computational tool is required for this purpose. While it is possible to perform community – or any other network descriptor – analysis in each layer separately, only by performing multilayer analysis it is possible to account for the continuity of communities – or centrality, influence, clusters, and so forth – over time, a key advantage that has no counterpart in other single-layer or aggregated approaches. This result, heavily based on the multilayer analysis of functional brain connectivity, suggests the existence of a trade-off between a region’s specialization and its capacity for flexible network reconfiguration [52] and highlights the power of this novel analytical framework to improve our understanding of the brain’s functional dynamics.

While brain activity during a single task can be studied by means of a temporal network, it has been recently shown that the networks corresponding to different tasks can be used to encode the layers of a multitask multilayer topology [53]. At variance with the temporal networks described above, where replicated nodes are interconnected across layers following the arrow of time (i.e., node *i* in the layer corresponding to snapshot τ is linked to node *i* in the layer(s) corresponding to τ^΄^ > τ), interconnectivity in multitask networks is categorical (i.e., node *i* in layer α is linked to all of its replicas in layers β ≠ α). Results from this research direction indicate that several inter-region temporal patterns observed at rest are preserved during different tasks, suggesting the existence of a primary intrinsic functional network architecture – similar to the one observed in a resting state – that is enriched by a secondary task-dependent functional connectivity [53].

## Structural and functional decomposition

Understanding the interplay between brain structure, function, and dynamics is a longstanding challenge [2–54]. The novel multilayer framework provides a unique opportunity to study, simultaneously, structural and functional information, and, in fact, it has been recently used for this purpose [59,60].

The first study concerns motifs, specific subgraphs of reduced size (generally 3 or 4 nodes), that play a fundamental role in the stability of the underlying system and several functions [61]. The significance of a motif is usually estimated by its occurrence with respect to a null model of the network. While the relationship between structural and functional brain motifs has been studied in the past [62], in Battison et al. (2016), the authors have exploited the recent mathematical advances in network analysis to investigate multiplex motifs [59,63].

In their setup, each multiplex network consists of two layers: one reflecting anatomical connectivity – inferred from diffusion magnetic resonance imaging – and one encoding functional relationships – inferred from fMRI – among the brain regions of healthy subjects. In this context, multiplex motifs are potentially more informative than their single-layer (either structural or functional) counterparts taken separately because a larger number of configurations, accounting for both layers simultaneously, is considered. The results indicate that when a physical connection between different brain regions coexists with a non-trivial positive correlation in their activities, the corresponding motif is statistically significant; i.e., it occurs more frequently than random expectation. As a consequence, this works provides further quantitative support to the hypothesis that functional connectivity is non-trivially constrained by brain architecture.

In the same spirit, another study explored the relationship between the structure and function of the Macaque cortical network [60]. In this case, the functional layer has been derived from simulated neural activity, whereas structural information is provided by anatomical connectivity. From the study of multiplex clustering, involving triangles of nodes on the two layers, the authors have investigated the emergence of functional connections that have no structural counterpart and the dependence of the multiplex network on the neural dynamical regime.

## Conclusion

Increasing evidence shows that our understanding of the human brain cannot prescind from using more complex multi-scale and multilayer models than a decade ago. The new models have to account for the hierarchical organization of the brain in both spatial and temporal dimensions, as well as its functional organization changes across temporal and frequency domains, while interplaying with the underlying structure. The recent advances in network science led to the development of a powerful mathematical framework for multilayer networks [7], topologies able to account for the simultaneous existence of different types of relationships between system’s units and their variation over time [5,6,11].

The present epoch is mature enough for multilayer analysis of the human brain to investigate the functional role of brain regions in different domains. While the field is still in its infancy, intense research activity is ongoing, based on advanced mathematical models to represent structural and functional connectivity, their evolution over time and their interdependence. Network science is just coming out of the multilayer revolution, which triggered hundreds of applications in all disciplines, from life sciences to humanities, in a few years. While there are still many theoretical challenges to tackle, such as the definition of appropriate null models to compare against the connectivity of empirical multilayer systems [64–67], the outcome of such a revolution already provides several computational tools to identify key units in multilayer systems [7,32,34,35,68,69], determine their organization in modules [8,9,70,71], reduce connectivity into simpler architectures [31], and discover the hierarchical organization of layers [72].

The application of some of these tools to the analysis of the human brain provides exciting novel insights about the brain’s structure and function. Nevertheless, from a methodological point of view, it is still a challenge to define a physical meaning for inter-layer connectivity, beyond purely mathematical or computational arguments. For instance, in multiplex networks representing multimodal connectivity or structural-functional relationships, as well as in time-varying networks, nodes replicated across different layers are linked with each other: in the former, all the replicas are interconnected, whereas in the latter only replicas corresponding to subsequent temporal snapshots are connected in order to preserve the underlying causal structure of the data. However, the weight to assign to these inter-layer connections is a free parameter, as in the case of frequency-based decompositions (in this last case, a partial solution might be given by the analysis of cross-frequency correlations).

In the future, we expect more complex structural and dynamical models able to account for several types of information simultaneously. Such models will incorporate multivariate information from different domains, e.g., space, time, and frequency, across different scales, from the cellular level to entire brain regions, with the ultimate goal of shedding light on how the interplay between structure and dynamics is related to brain diseases and gives rise to cognition.

## Competing interests

The author declares that he has no competing interests.

## Author contributions

The author conceived the idea for this correspondence, conceptualized and wrote this article.

## Supplementary Material

GIGA-D-16-00168_Original_Submission.pdf

GIGA-D-16-00168_Revision_1.pdf

Response_to_Reviewer_Comments_Original_Submission.pdf

Reviewer_1_Original_Submission_(Attachement_).pdf

Reviewer_1_Report_(Original_Submission).pdf

Reviewer_2_Report_(Original_Submission).pdf

Reviewer_3_Report_(Original_Submission).docx

Reviewer_3_Report_(Original_Submission).pdf
