# Supplementary material for: Multilayer modeling and analysis of human brain networks
Source: Gigascience. 2017 Feb 6;6(5):1–8. doi: 10.1093/gigascience/gix004 (PMC5437946; doi:10.1093/gigascience/gix004)
Supplement: GIGA-D-16-00168_Original_Submission.pdf [file gix004_GIGA-D-16-00168_Original_Submission.pdf]

Manuscript Click here to download Manuscript muxbrain.tex

REVIEW

The rise of multilayer modeling and analysis of human brain networks

Manlio De Domenico

Abstract

Understanding how the human brain is structured, and how its architecture is related to the function, is of paramount importance for a variety of applications, including, but not limited to, new ways to prevent, deal with and cure brain diseases, such as Alzheimer's or Parkinson's, and psychiatric disorders, such as Schizophrenia. The recent advances in structural and functional neuroimaging, together with the increasing attitude to interdisciplinary approaches involving computer science, mathematics and physics, are fostering interesting results from computational neuroscience, that are quite often based on the analysis of complex network representation of human brain. In the last years, this representation experienced a theoretical and computational revolution that are breaching neuroscience, allowing to cope with the increasing complexity of human brain across multiple scales and in multiple dimensions, and to model structural and functional connectivity from new perspectives, often combined with each other. In this work, we will review the main achievements obtained from interdisciplinary research based on magnetic resonance imaging and establishing, *de facto*, the birth of multilayer network analysis and modeling of human brain.

Keywords: multilayer networks; functional connectivity; structural reducibility; versatility

Background

Brain networks provide a map of the complex organization, either structural or functional, of its units. In the last decades, several experimental measurements, based on electro-encephalography (EEG), magneto-encephalography (MEG), diffusion tensor imaging (DTI), structural and functional magnetic resonance imaging (fMRI), have been carried on to explore such an organization [1, 2].

In this context, networks consist of brain regions (i.e., the nodes) and their structural or functional connection patterns (i.e., the edges) obtained by evaluating cross-correlation or more sophisticated similarity measures in space, time and frequency domains. This network modeling approach successfully unveiled interesting features such as small-worldness – where the underlying topology is highly locally clustered and the presence of long-range connections dramatically reduce the distance between units – modular and rich-club organization – where the underlying topology can be coarse-grained and described as a network of modules, with highly-connected units tending to be connected each other more frequently than random expectation. The success of network mapping increased, in parallel, the need for novel methodologies devoted to unravel the structure and the function of the brain at multiple spatial and temporal scales [3].

However, the lack of an appropriate mathematical framework for the representation and analysis of multivariate connectivity data forced many studies to neglect, disregard or aggregate available information, in order to cope with the high amount of underlying complexity.

More recently, researchers explored the possibility to study the human brain without necessarily either throwing out or aggregating the data deluge available nowadays. The most promising approach is to use multilayer networks (see Refs. [4, 5] for a thorough review), recently developed to provide a mathematical framework [6] to model and analyze complex data with multivariate and multi-scale information. Recent results from this research direction are exciting and provide new insights about our understanding of structure and function of the human brain.

Multilayer network representation of human brain

A multilayer network consists of several distinct classical networks, each one encoding a specific type of information about the system. In the following, we will

Correspondence: manlio.dedomenico@urv.cat
Departament d'Enginyeria Informàtica i Matemàtiques, Universitat Rovira i Virgili, Av.da Països Catalans, 26, 43004 Tarragona, Spain
Full list of author information is available at the end of the article

briefly discuss different types of multilayer brain networks where layers' connectivity, measured with respect to a specific definition of similarity (e.g., cross-correlation, spectral coherence, and so forth so on) might encode i) activity in different frequency bands; ii) time-varying activity; iii) activity with respect to different tasks; iv) structural and functional connectivity.

While standard networks can be represented by adjacency matrices, indicating the presence and the intensity of connections among system's units, multilayer networks requires higher-order matrices, i.e. tensors, to be appropriately represented [6] (see Figure 1a). In general, the components of the multilayer adjacency tensor of N nodes and L layers are indicated by $M_{j\beta}^{i\alpha}$ and encode the connectivity between unit i in layer α and unit j in layer β, with $i, j = 1, 2, ..., N$. For instance, intra-layer connectivity in the $\alpha-$th layer is given by the entries $M_{j\alpha}^{i\alpha}$. A standard approach is based on flattening this rank-4 tensor into a rank-2 tensor, named supra-adjacency matrix, with a block structure where diagonal blocks encode intra-layer connectivity and off-diagonal blocks encode inter-layer connectivity (Figure 1b).

The tensorial representation of multilayer networks allows to develop a powerful mathematical framework to extend traditional complex network analysis such as detection of modular super-units [7, 8] and identification of most central units [9]. The majority of such tools is based on the analysis of how information spreads through the multilayer system (see Ref. [10] and references therein) and provides a suitable framework for the analysis of human brain.

Frequency-based decomposition

The functional connectivity of the brain is usually obtained by measuring a specific type of physical signal from different regions and then comparing pairwise signals by means of some similarity measure (e.g., cross-correlation, transfer of entropy, spectral coherence, so forth so on). If the similarity between two signals is statistically significant, a functional link is considered between the corresponding brain regions. Many studies differ in the type of signal they measure and the statistical methodology adopted to build the functional network, but they all share the approach described above.

In the case of fMRI, signals are filtered and components between 0.01 and 0.1 Hz are usually kept [11, 12, 13] (see Ref. [14] for a review). The choice of the frequency band might have deep impact on the functional representation of the brain. In fact, standard methodologies do not distinguish the contributions coming from different frequency bands, considering only one specific range. The resulting network provides a functional map of the brain and allows to identify special regions which act as hubs, i.e. units either with larger connectivity than others or with strategic functions which maximize the information flow through them [15, 16, 17, 18] (see Ref. [14] for a review).

Given their functional importance, hubs mediate interactions among other regions and might favor the brain's integrated operation. They are generally identified by centrality descriptors [19] and they are of particular interest in many applications [20, 21, 22, 23]. Recent studies have shown that the importance of each region is subjected to dramatic changes depending on the frequency cuts [24] and that hubs might be very different when functional connectivity is measured in different frequency bands [25]. These results, together with previous findings concerning the importance of topological information measured from components above 0.1 Hz [26, 27, 28], suggest that a novel framework for modeling and analysis of human brain functional connectivity is required.

The new framework must be able to consider functional information from different frequency bands, simultaneously: in practice, for each band it is sufficient to build a functional network and then to analyze the resulting system as a whole. Multilayer networks provide the mathematical background [6] for this purpose. In this new framework, each region of the brain is mapped into a network node and replicated across all layers, encoding frequency bands, where they are connected with other nodes by means of functional links – corresponding to significant correlations in a specific frequency band. The methodology is summarized in the top panels of Fig. 2.

Nodes are interconnected with their replicas – also known as *state nodes* – across layers and the weight of these links is, in general, a free parameter which must be estimated from the data or by maximizing a specific cost function [29]. From the perspective of a single unit, generally named *physical node*, inter-layer connectivity between the corresponding state nodes constitutes a clique.

The first question to answer is to which extent such an enriched representation of functional connectivity is more valuable than other aggregated (or less rich) representations. The answer has been recently given in Ref. [29], where it has been shown that each functional layer – in a range between 0.01 Hz and 0.25 Hz, in steps of 0.02 Hz – provides unique information and should be neither aggregated with other layers nor neglected. The result, is based on the analysis of structural reducibility [30], a modern technique grounded on information entropy.

The irreducibility of the multilayer functional representation of human brain raises the necessity for multilayer analysis of the underlying architecture and a few first results have been recently reported about the identification of hubs. In other contexts, it has been shown that hubs in a multilayer network might be dramatically different from hubs in each layer of the system [31]. An intuitive example is given in the following. Let us consider a two-layer system where a certain node is in the periphery of both networks, and let us consider that such a node is the only one in common to the two layers. It is clear that this node is crucial for the exchange of information between the two layers and, as a consequence, it will be most central with respect to this criterion, even if in each layer, considered separately, it is peripheral and it would be the less central[1].

The multilayer analysis of brain's regions centrality reveals that hubs are, in general, different from the hubs identified by standard methodologies based on single-layer network analysis. The most surprising finding is that such hubs can be used to distinguish, with high accuracy, the brain of a schizophrenic patient from a healthy brain in resting state [30], thus improving our understanding of schizophrenia and opening the door to the analysis of other brain disorders within the same framework.

Magnetoencephalography (MEG) has been recently used in a similar spirit, with layers encoding the connectivity between neural oscillations within four frequency bands, namely alpha (8 Hz–13 Hz), beta (13 Hz–30 Hz), low gamma (30 Hz–50 Hz) and high gamma (50 Hz–100 Hz). In this context, the mean connection strength – averaged across the network where the functional connectivity between schizophrenic patients and controls differs most – has been used to gain new insights about within and between oscillatory frequencies [35]. Two regimes of multilayer network behavior have been identified in a system with five layers (bands 1 Hz–4 Hz, 4 Hz–8 Hz, 8 Hz–13 Hz, 13 Hz–30 Hz and 30 Hz–48 Hz): in the first regime layers are independent, while in the second regime they are highly dependent. Results suggest that healthy human brain operates at the transition point between these two regimes [36].

These studies provide evidence for and support the hypothesis that functional layers do not act as independent entities, suggesting the existence of mechanisms for integration and segregation of brain activity within and across different frequency bands. Very recently, a mechanistic model for this process has been proposed [37]. The authors have compared the performance of two models: in the model A, each brain region generates oscillations in a single frequency; in model B, each brain region can generate oscillations in multiple frequency bands. The model B, named multi-frequency model, does not take into account cross-frequency interactions but it still outperforms single-frequency model in reproducing empirical MEG data [37].

[1] Here, information exchange can be modeled by bits diffusing through the system either along random walks [32] or shortest paths [33, 34] between two endpoints.

Time-varying network model and task-based decomposition

Instead of building functional layers in the frequency domain, it might be desirable to consider the brain activity in the time domain, because temporal changes and their mapping might be biologically meaningful. It is worth noting that historically this was, in fact, the first multilayer approach to the analysis of brain networks, even when a formal theory for this type of structure was not yet available [38].

Usually, the measured BOLD activity is divided into a series of time windows named snapshots, – which can be overlapping or not – and a pairwise measure of correlation between regions of interest is calculated to build a functional network for each snapshot. The resulting network is a multilayer graph where each layer corresponds to a functional snapshot of brain activity. This approach has the advantage of building a static backbone of the underlying functional dynamic of human brain that can be used, for instance, to better understand how it operates during specific tasks or on the onset of an epileptic seizure. In this regard, multilayer networks describing how functional connectivity changes across time provide a richer framework than traditional approaches [39]. In this framework, state nodes are interconnected only with their subsequent replicas, like in a chain. This methodology, summarized in the bottom panels of Fig. 2, has opened the door to several studies and triggered the development of novel theoretical measures to identify the most influent brain regions during learning [40] and how they cluster together in functional modules [41], to cite some of them.

The multilayer model for time-varying networks can be used to explore the role of functional fluctuations while in resting state or performing specifying activities (see Ref. [3] for an up-to-date review), where in the latter case one defines a task-based representation of brain activity [41, 42]. This type of decomposition is of particular interest because it is possible to map the reconfiguration of brain regions' correlated activity between different tasks or during a learning process [43, 44].

Besides the variety of its applications, very recently, this novel framework has been used to better characterize high-level language processing in humans by using fMRI data from 22 human subjects, asked to perform a language comprehension task. While it is known that the activity of left frontal, temporal, and parietal cortices is very correlated – constituting a functional system – when an individual is performing a naturalistic language comprehension task or she is resting, it is still poorly understood how they become part of such an integrated functional system. By identifying functional modules within the multilayer framework, involving the generalization of classical modularity maximization to the multilayer domain [7], it has been shown that a stable core of mutually co-activating brain regions emerges mainly in the left hemisphere, whereas a periphery of brain regions is developed in the right hemisphere, while co-activating with different regions at different times. One might ask if it is required to use such a complicated computational tool for this purpose. While it is possible to perform community – or any other network descriptor – analysis in each layer separately, only by performing multilayer analysis it is possible to account for the continuity of communities – or centrality, influence, clusters, and so forth so on – over time, a key advantage that has no counterpart in other single-layer or aggregated approaches. This result, heavily based on the multilayer analysis of functional brain connectivity, suggests the existence of trade-off between a region's specialization and its capacity for flexible network reconfiguration [45] and highlights the power of this novel analytical framework to improve our understanding of brain's functional dynamics.

Structural and functional decomposition

Understanding the interplay between brain structure, function and dynamics is a longstanding challenge [46, 47, 48, 49, 50, 2]. The novel multilayer framework provides a unique opportunity to study, simultaneously, structural and functional information and, in fact, it has been recently used for this purpose [51, 52].

The first study concerns motifs, specific subgraphs of reduced size (generally 3 or 4 nodes) that play a fundamental role for the stability of the underlying system and several functions [53]. The significance of a motif is usually estimated by its occurrence with respect to a null model of the network. While the relationship between structural and functional brain motifs has been studied in the past [54], in Ref. [51] the authors have exploited the recent mathematical advances in network analysis to investigate multiplex motifs [55].

In their setup, each multiplex network consists of two layers: one reflecting anatomical connectivity – inferred from Diffusion Magnetic Resonance Imaging – and one encoding functional relationships – inferred from functional Magnetic Resonance Imaging – among the brain regions of healthy subjects. In this context, multiplex motifs are potentially more informative than their single-layer (either structural or functional) counterparts taken separately, because a larger number of configurations, accounting for both layers simultaneously, is considered. The results indicate that when a physical connection between different brain regions coexists with a non-trivial positive correlation in their activities, the corresponding motif is statistically significant, i.e. it occurs more frequently than random expectation. As a consequence, this works provides further quantitative support to the hypothesis that functional connectivity is non-trivially constrained by brain architecture.

In the same spirit, another study explored the relationship between structure and function the Macaque cortical network [52]. In this case, the functional layer has been derived from simulated neural activity, whereas structural information is provided by anatomical connectivity. From the study of multiplex clustering, involving triangles of nodes on the two layers, the authors have investigated the emergence of functional connections that have no structural counterpart and the dependence of the multiplex network on the neural dynamical regime.

Conclusion

Increasing evidences show that our understanding of human brain cannot prescind from using more complex multi-scale and multilayer models than a decade ago. The new models have to account for the hierarchical organization of the brain in both spatial and temporal dimensions, as well as its functional organization changes across temporal and frequency domains, while interplaying with the underlying structure. The recent advances in network science led to the development of a powerful mathematical framework for multilayer networks [6], topologies able to account for the simultaneous existence of different types of relationships between system's units and their variation over time [4, 5, 10].

The present epoch is mature enough for multilayer analysis of human brain, to investigate the functional role of brain regions in different domains [56, 6, 33, 34, 31], their organization in modules [7, 8, 57, 58] and the reducibility of their connectivity into simpler architectures [30].

Competing interests
The author declares that he has no competing interests.

Author's contributions
The author conceived the idea for this correspondence, conceptualized and wrote this article.

Acknowledgements
The author acknowledges financial support from the Spanish program Juan de la Cierva (IJCI-2014-20225).

References

1. Bullmore, E., Sporns, O.: Complex brain networks: graph theoretical analysis of structural and functional systems. Nature Reviews Neuroscience **10**(3), 186–198 (2009)
2. Park, H.-J., Friston, K.: Structural and functional brain networks: from connections to cognition. Science **342**(6158), 1238411 (2013)
3. Betzel, R.F., Bassett, D.S.: Multi-scale brain networks. To appear in NeuroImage (arXiv:1608.08828) (2016)
4. Kivelä, M., Arenas, A., Barthelemy, M., Gleeson, J.P., Moreno, Y., Porter, M.A.: Multilayer networks. Journal of complex networks **2**(3), 203–271 (2014)
5. Boccaletti, S., Bianconi, G., Criado, R., Del Genio, C.I., Gómez-Gardeñes, J., Romance, M., Sendiña-Nadal, I., Wang, Z., Zanin, M.: The structure and dynamics of multilayer networks. Physics Reports **544**(1), 1–122 (2014)
6. De Domenico, M., Solé-Ribalta, A., Cozzo, E., Kivelä, M., Moreno, Y., Porter, M.A., Gómez, S., Arenas, A.: Mathematical formulation of multilayer networks. Physical Review X **3**(4), 041022 (2013)
7. Mucha, P.J., Richardson, T., Macon, K., Porter, M.A., Onnela, J.-P.: Community structure in time-dependent, multiscale, and multiplex networks. science **328**(5980), 876–878 (2010)
8. De Domenico, M., Lancichinetti, A., Arenas, A., Rosvall, M.: Identifying modular flows on multilayer networks reveals highly overlapping organization in interconnected systems. Physical Review X **5**(1), 011027 (2015)
9. De Domenico, M., Solé-Ribalta, A., Omodei, E., Gómez, S., Arenas, A.: Ranking in interconnected multilayer networks reveals versatile nodes. Nature communications **6**, 6868 (2015)
10. De Domenico, M., Granell, C., Porter, M.A., Arenas, A.: The physics of spreading processes in multilayer networks. Nature Physics **12**, 901 (2016)
11. Cordes, D., Haughton, V.M., Arfanakis, K., Carew, J.D., Turski, P.A., Moritz, C.H., Quigley, M.A., Meyerand, M.E.: Frequencies contributing to functional connectivity in the cerebral cortex in ?resting-state? data. American Journal of Neuroradiology **22**(7), 1326–1333 (2001)
12. Cordes, D., Haughton, V., Carew, J.D., Arfanakis, K., Maravilla, K.: Hierarchical clustering to measure connectivity in fmri resting-state data. Magnetic resonance imaging **20**(4), 305–317 (2002)
13. Fox, M.D., Raichle, M.E.: Spontaneous fluctuations in brain activity observed with functional magnetic resonance imaging. Nature Reviews Neuroscience **8**(9), 700–711 (2007)
14. Fallani, F.D.V., Richiardi, J., Chavez, M., Achard, S.: Graph analysis of functional brain networks: practical issues in translational neuroscience. Phil. Trans. R. Soc. B **369**(1653), 20130521 (2014)
15. Van Den Heuvel, M.P., Pol, H.E.H.: Exploring the brain network: a review on resting-state fmri functional connectivity. European Neuropsychopharmacology **20**(8), 519–534 (2010)
16. Poldrack, R.A., Farah, M.J.: Progress and challenges in probing the human brain. Nature **526**(7573), 371–379 (2015)
17. Achard, S., Salvador, R., Whitcher, B., Suckling, J., Bullmore, E.: A resilient, low-frequency, small-world human brain functional network with highly connected association cortical hubs. The Journal of Neuroscience **26**(1), 63–72 (2006)
18. Power, J.D., Schlaggar, B.L., Lessov-Schlaggar, C.N., Petersen, S.E.: Evidence for hubs in human functional brain networks. Neuron **79**(4), 798–813 (2013)
19. Boccaletti, S., Latora, V., Moreno, Y., Chavez, M., Hwang, D.-U.: Complex networks: Structure and dynamics. Physics reports **424**(4), 175–308 (2006)
20. Sporns, O., Honey, C.J., Kötter, R.: Identification and classification of hubs in brain networks. PloS one **2**(10), 1049–1049 (2007)
21. Lynall, M.-E., Bassett, D.S., Kerwin, R., McKenna, P.J., Kitzbichler, M., Muller, U., Bullmore, E.: Functional connectivity and brain networks in schizophrenia. The Journal of Neuroscience **30**(28), 9477–9487 (2010)
22. Rubinov, M., Sporns, O.: Complex network measures of brain connectivity: uses and interpretations. Neuroimage **52**(3), 1059–1069 (2010)
23. Zuo, X.-N., Ehmke, R., Mennes, M., Imperati, D., Castellanos, F.X., Sporns, O., Milham, M.P.: Network centrality in the human functional connectome. Cerebral cortex **22**(8), 1862–1875 (2012)
24. Thompson, W.H., Fransson, P.: The frequency dimension of fmri dynamic connectivity: Network connectivity, functional hubs and integration in the resting brain. NeuroImage **121**, 227–242 (2015)
25. Sasai, S., Homae, F., Watanabe, H., Sasaki, A., Tanabe, H., Sadato, N., Taga, G.: Frequency-specific network topologies in the resting human brain. Frontiers in human neuroscience **8**, 1022 (2014)
26. Bassett, D.S., Meyer-Lindenberg, A., Achard, S., Duke, T., Bullmore, E.: Adaptive reconfiguration of fractal small-world human brain functional networks. PNAS **103**(51), 19518–19523 (2006)
27. Supekar, K., Menon, V., Rubin, D., Musen, M., Greicius, M.D., *et al.*: Network analysis of intrinsic functional brain connectivity in alzheimer?s disease. PLoS Comput Biol **4**(6), 1000100 (2008)
28. Chavez, M., Valencia, M., Navarro, V., Latora, V., Martinerie, J.: Functional modularity of background activities in normal and epileptic brain networks. Physical Review Letters **104**(11), 118701 (2010)
29. De Domenico, M., Sasai, S., Arenas, A.: Mapping multiplex hubs in human functional brain networks. Frontiers in Neuroscience **10**, 326 (2016)
30. De Domenico, M., Nicosia, V., Arenas, A., Latora, V.: Structural reducibility of multilayer networks. Nature communications **6**, 6864 (2015)
31. De Domenico, M., Solé-Ribalta, A., Omodei, E., Gómez, S., Arenas, A.: Ranking in interconnected multilayer networks reveals versatile nodes. Nature Communications **6**, 6868–6868 (2015)
32. De Domenico, M., Solé-Ribalta, A., Gómez, S., Arenas, A.: Navigability of interconnected networks under random failures. Proceedings of the National Academy of Sciences **111**(23), 8351–8356 (2014)
33. Battiston, F., Nicosia, V., Latora, V.: Structural measures for multiplex networks. Physical Review E **89**(3), 032804 (2014)
34. Solé-Ribalta, A., De Domenico, M., Gómez, S., Arenas, A.: Centrality rankings in multiplex networks. In: Proceedings of the 2014 ACM Conference on Web Science, pp. 149–155 (2014). ACM
35. Brookes, M.J., Tewarie, P.K., Hunt, B.A., Robson, S.E., Gascoyne, L.E., Liddle, E.B., Liddle, P.F., Morris, P.G.: A multi-layer network approach to meg connectivity analysis. NeuroImage **132**, 425–438 (2016)
36. Tewarie, P., Hillebrand, A., van Dijk, B.W., Stam, C.J., O'Neill, G.C., Van Mieghem, P., Meier, J.M., Woolrich, M.W., Morris, P.G., Brookes, M.J.: Integrating cross-frequency and within band functional networks in resting-state meg: A multi-layer network approach. NeuroImage **142**, 324–336 (2016)
37. Deco, G., Cabral, J., Woolrich, M., Stevner, A., van Hartevelt, T., Kringelbach, M.: Single or multi-frequency generators in on-going brain activity: a mechanistic whole-brain model of empirical meg data. bioRxiv:084103 (2016). doi:10.1101/084103. http://biorxiv.org/content/early/2016/11/04/084103.full.pdf
38. Honey, C.J., Kötter, R., Breakspear, M., Sporns, O.: Network structure of cerebral cortex shapes functional connectivity on multiple time scales. Proceedings of the National Academy of Sciences **104**(24), 10240–10245 (2007)
39. Holme, P., Saramäki, J.: Temporal networks. Physics reports **519**(3), 97–125 (2012)
40. Mantzaris, A.V., Bassett, D.S., Wymbs, N.F., Estrada, E., Porter, M.A., Mucha, P.J., Grafton, S.T., Higham, D.J.: Dynamic network centrality summarizes learning in the human brain. Journal of Complex Networks **1**(1), 83–92 (2013)
41. Bassett, D.S., Wymbs, N.F., Porter, M.A., Mucha, P.J., Carlson, J.M., Grafton, S.T.: Dynamic reconfiguration of human brain networks during learning. PNAS **108**(18), 7641–7646 (2011)
42. Bassett, D.S., Wymbs, N.F., Rombach, M.P., Porter, M.A., Mucha, P.J., Grafton, S.T.: Task-based core-periphery organization of human brain dynamics. PLoS Comput Biol **9**(9), 1003171 (2013)
43. Mattar, M.G., Cole, M.W., Thompson-Schill, S.L., Bassett, D.S.: A functional cartography of cognitive systems. PLoS Comput Biol **11**(12), 1004533 (2015)
44. Bassett, D.S., Yang, M., Wymbs, N.F., Grafton, S.T.: Learning-induced autonomy of sensorimotor systems. Nature

Neuroscience **18**(5), 744–751 (2015)
45. Chai, L.R., Mattar, M.G., Blank, I.A., Fedorenko, E., Bassett, D.S.: Functional network dynamics of the language system. Cerebral Cortex **26**, 4148–4159 (2016)
46. Cohen, J.D., Perlstein, W.M., Braver, T.S., Nystrom, L.E., Noll, D.C., Jonides, J., Smith, E.E., *et al.*: Temporal dynamics of brain activation during a working memory task. Nature **386**(6625), 604–608 (1997)
47. Sporns, O., Chialvo, D.R., Kaiser, M., Hilgetag, C.C.: Organization, development and function of complex brain networks. Trends in cognitive sciences **8**(9), 418–425 (2004)
48. Draganski, B., Gaser, C., Kempermann, G., Kuhn, H.G., Winkler, J., Büchel, C., May, A.: Temporal and spatial dynamics of brain structure changes during extensive learning. The Journal of Neuroscience **26**(23), 6314–6317 (2006)
49. Rubinov, M., Sporns, O., van Leeuwen, C., Breakspear, M.: Symbiotic relationship between brain structure and dynamics. BMC neuroscience **10**(1), 1 (2009)
50. Deisseroth, K., Schnitzer, M.J.: Engineering approaches to illuminating brain structure and dynamics. Neuron **80**(3), 568–577 (2013)
51. Battiston, F., Nicosia, V., Chavez, M., Latora, V.: Multilayer motif analysis of brain networks. To appear in Chaos (arXiv:1606.09115) (2016)
52. Crofts, J. J., Forrester, M., O'Dea, R. D.: Structure-function clustering in multiplex brain networks. EuroPhysics Letters **116**(1), 18003 (2016)
53. Milo, R., Shen-Orr, S., Itzkovitz, S., Kashtan, N., Chklovskii, D., Alon, U.: Network motifs: simple building blocks of complex networks. Science **298**(5594), 824–827 (2002)
54. Sporns, O., Kötter, R.: Motifs in brain networks. PLoS Biol **2**(11), 369 (2004)
55. Wernicke, S., Rasche, F.: Fanmod: a tool for fast network motif detection. Bioinformatics **22**(9), 1152–1153 (2006)
56. Tang, J., Musolesi, M., Mascolo, C., Latora, V., Nicosia, V.: Analysing information flows and key mediators through temporal centrality metrics. In: Proceedings of the 3rd Workshop on Social Network Systems, p. 3 (2010). ACM
57. Peixoto, T.P.: Inferring the mesoscale structure of layered, edge-valued, and time-varying networks. Physical Review E **92**(4), 042807 (2015)
58. Valles-Catala, T., Massucci, F.A., Guimera, R., Sales-Pardo, M.: Multilayer stochastic block models reveal the multilayer structure of complex networks. Physical Review X **6**(1), 011036 (2016)

Figures

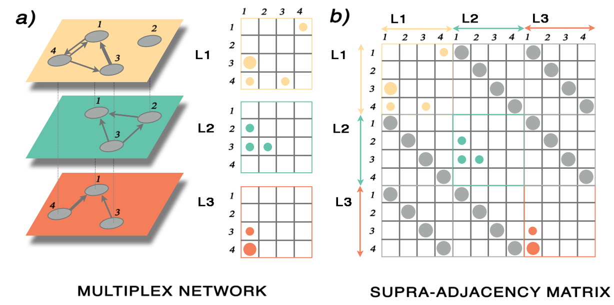

Figure 1 Multilayer network representation. **(a)** A multilayer network consists of different networks encoded by layers, each one represented by a (possibly directed and weighted) adjacency matrix. **(b)** The rank-4 multilayer adjacency tensor, representing intra- and inter-layer connectivity is generally flattened by matricization to a rank-2 tensor, generally known as supra-adjacency matrix, without loss of information.

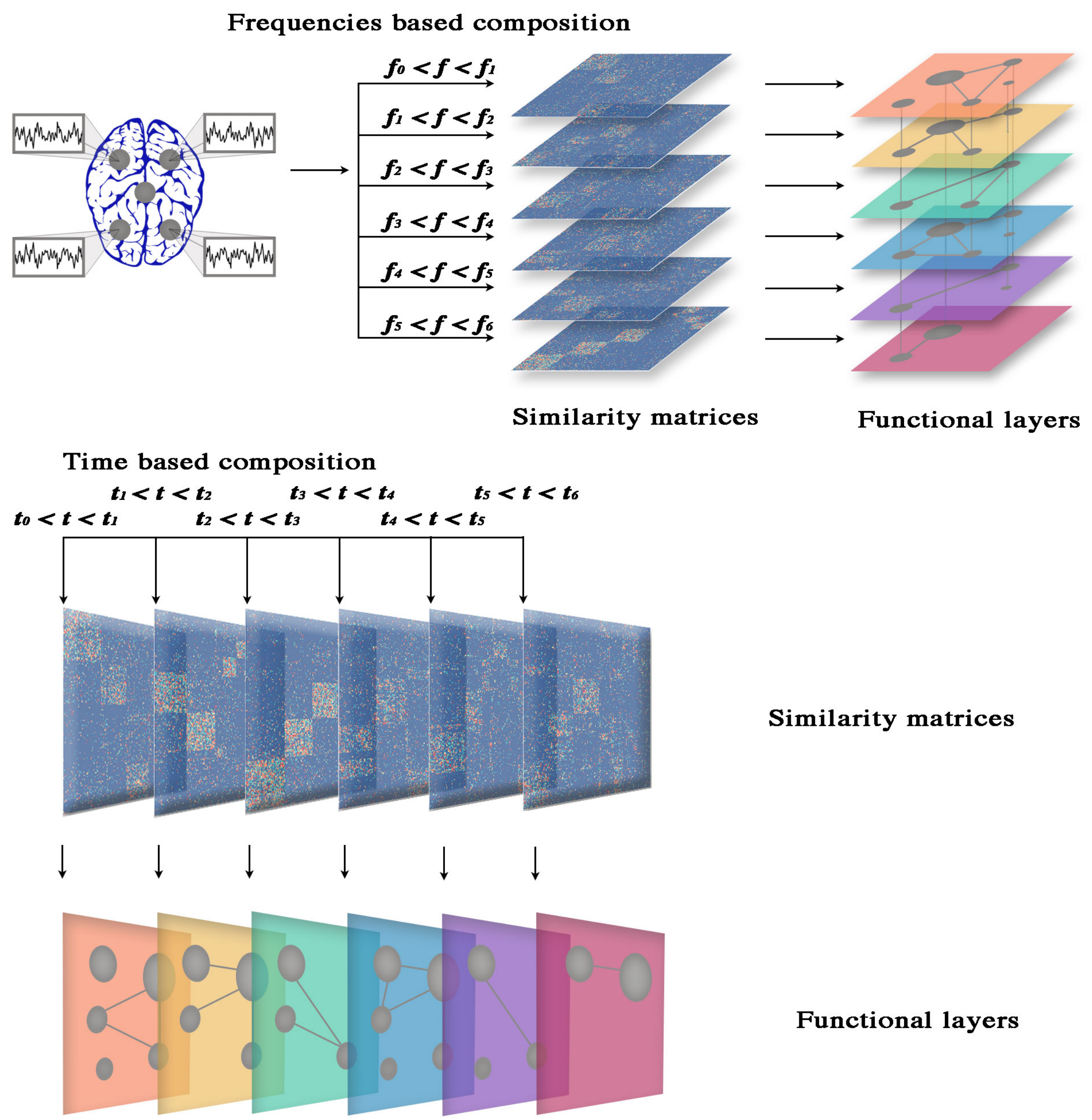

Figure 2 Multilayer functional brain. Top panels: brain activity is measured in different regions and signals are decomposed in the frequency domain. The frequency domain consists of (possibly overlapping) frequency bands and, for each band, coherence – or other similarity descriptors – is measured between all pairs of regions. A similarity matrix is built for each frequency domain and statistical analysis of significance is used to map each matrix into a functional network, constituting a functional layer of the overall multilayer system. **Bottom panels:** in this case, signals are decomposed in the time domain, which consists of (possibly overlapping) consecutive temporal snapshots. A similarity matrix is calculated for each snapshot and the corresponding functional layer is built.
