# Supplementary material for: Multilayer modeling and analysis of human brain networks
Source: Gigascience. 2017 Feb 6;6(5):1–8. doi: 10.1093/gigascience/gix004 (PMC5437946; doi:10.1093/gigascience/gix004)
Supplement: GIGA-D-16-00168_Revision_1.pdf [file gix004_GIGA-D-16-00168_Revision_1.pdf]

Manuscript Click here to download Manuscript muxbrain.tex

REVIEW

Multilayer modeling and analysis of human brain networks

Manlio De Domenico

Abstract

Understanding how the human brain is structured, and how its architecture is related to the function, is of paramount importance for a variety of applications, including, but not limited to, new ways to prevent, deal with and cure brain diseases, such as Alzheimer's or Parkinson's, and psychiatric disorders, such as Schizophrenia. The recent advances in structural and functional neuroimaging, together with the increasing attitude to interdisciplinary approaches involving computer science, mathematics and physics, are fostering interesting results from computational neuroscience, that are quite often based on the analysis of complex network representation of human brain. In the last years, this representation experienced a theoretical and computational revolution that are breaching neuroscience, allowing to cope with the increasing complexity of human brain across multiple scales and in multiple dimensions, and to model structural and functional connectivity from new perspectives, often combined with each other. In this work, we will review the main achievements obtained from interdisciplinary research based on magnetic resonance imaging and establishing, *de facto*, the birth of multilayer network analysis and modeling of human brain.

Keywords: multilayer networks; functional connectivity; structural reducibility; versatility

Background

Brain networks provide a map of the complex organization, either structural or functional, of its units. In the last decades, several experimental measurements, based on electro-encephalography (EEG), magneto-encephalography (MEG), diffusion tensor imaging (DTI), structural and functional magnetic resonance imaging (fMRI), have been carried on to explore such an organization [1, 2].

In this context, networks consist of brain regions (i.e., the nodes) and their structural or functional connection patterns (i.e., the edges) obtained by evaluating cross-correlation or more sophisticated similarity measures in space, time and frequency domains.

Structural networks usually represent an anatomical parcellation of the brain where links among neurons or regions, encoding physical connections, are obtained from MRI, DTI or histological data. In DTI, one of the widest adopted techniques, the diffusion of water molecules and the result of their interactions with tissues are measured with high accuracy, allowing to reconstruct nerve fibers and to map the human brain in three dimensions with exceptional resolution.

Correspondence: manlio.dedomenico@urv.cat
Departament d'Enginyeria Informàtica i Matemàtiques, Universitat Rovira i Virgili, Av.da Països Catalans, 26, 43004 Tarragona, Spain
Full list of author information is available at the end of the article

The functional connectivity of the brain is usually obtained by measuring a specific type of physical signal (e.g., blood-oxygen-level dependent – i.e., BOLD – contrast as in fMRI or magnetic field as in MEG) from different regions and then comparing pairwise signals by means of some similarity measure (e.g., cross-correlation, transfer of entropy, spectral coherence, so forth so on). If the similarity between two signals is statistically significant, a functional link is considered between the corresponding brain regions. Many studies differ in the type of signal they measure and the statistical methodology adopted to build the functional network, but they all share the approach described above.

Network modeling approaches successfully unveiled interesting features such as small-worldness – where the underlying topology is highly locally clustered and the presence of long-range connections dramatically reduce the distance between units – modular and rich-club organization – where the underlying topology can be coarse-grained and described as a network of modules, with highly-connected units tending to be connected each other more frequently than random expectation. The success of network mapping increased, in parallel, the need for novel methodologies devoted to unravel the structure and the function of the brain at multiple spatial and temporal scales [3, 4]. However, the lack of an appropriate mathematical framework for

the representation and analysis of multivariate connectivity data forced many studies to neglect, disregard or aggregate available information, in order to cope with the high amount of underlying complexity.

More recently, researchers explored the possibility to study the human brain without necessarily either throwing out or aggregating the data deluge available nowadays. An important and promising approach is to use multilayer networks (see Refs. [5, 6] for a thorough review), recently developed to provide a mathematical framework [7] to model and analyze complex data with multivariate and multi-scale information. Recent results from this research direction are exciting and provide new insights about our understanding of structure and function of the human brain.

Multilayer network representation of human brain

A multilayer network consists of several distinct classical networks, each one encoding a specific type of information about the system. In the following, we will briefly discuss different types of multilayer brain networks where layers' connectivity, measured with respect to a specific definition of similarity (e.g., cross-correlation, spectral coherence, and so forth so on) might encode i) activity in different frequency bands; ii) time-varying activity; iii) activity with respect to different tasks; iv) structural and functional connectivity.

While standard networks can be represented by adjacency matrices, indicating the presence and the intensity of connections among system's units, multilayer networks requires higher-order matrices, i.e. tensors, to be appropriately represented [7] (see Figure 1a). In general, the components of the multilayer adjacency tensor of N nodes and L layers are indicated by $M_{j\beta}^{i\alpha}$ and encode the connectivity between unit i in layer α and unit j in layer β, with $i, j = 1, 2, ..., N$. For instance, intra-layer connectivity in the $\alpha-$th layer is given by the entries $M_{j\alpha}^{i\alpha}$. A standard approach is based on flattening this rank-4 tensor into a rank-2 tensor, named supra-adjacency matrix, with a block structure where diagonal blocks encode intra-layer connectivity and off-diagonal blocks encode inter-layer connectivity (Figure 1b).

The tensorial representation of multilayer networks allows us to develop a powerful mathematical framework to extend traditional complex network analysis such as detection of modular super-units [8, 9] and identification of most central units [10]. The majority of such tools is based on the analysis of how information spreads through the multilayer system (see Ref. [11] and references therein) and provides a suitable framework for the structural analysis of human brain.

While several classical network concepts have been successfully and satisfactorily extended to multilayer systems, approaches adopted to model human brain are mainly based on multiplex and interconnected multiplex topologies. In both models, the same node is usually replicated on more than one layer, where it exhibits different connectivity patterns depending on the information encoded by the layer. A multiplex topology is an edge-colored multigraph consisting of different layers that are not interconnected each other: $M_{j\beta}^{i\alpha} = 0$ for any $i, j = 1, 2, ..., N$ and $\alpha, \beta = 1, 2, ..., L$ (with $\alpha \neq \beta$), using the notation introduced before. An interconnected multiplex topology includes links across layers, although only the ones among node's replicas are allowed: $M_{j\beta}^{i\alpha} = 0$ for any $i \neq j$ and $\alpha \neq \beta$, whereas $M_{i\beta}^{i\alpha} \neq 0$ for $\alpha \neq \beta$ (as in Figure 1a). Other multilayer network models are possible, but they have found a few applications in neuroscience, if any.

It is worth remarking that one should be cautious in the choice of the network model to adopt for the analysis of human brain, if one is interested in exploiting the tensorial algebra developed to naturally extend the majority of classical network descriptors to the multilayer realm [7]. In fact, when interconnectivity is absent, analysis based on the multilayer adjacency tensor provides the same results of classical analysis of each layer separately.

In the following we will consider applications involving interconnected multiplex networks and we will refer to them as multiplex, for sake of simplicity.

Frequency-based decomposition

Frequency-based decomposition is an approach that provides a multilayer functional representation of human brain. In the case of fMRI, signals are filtered and components between 0.01 and 0.1 Hz are usually kept [12, 13, 14] (see Ref. [15] for a review). The choice of the frequency band might have deep impact on the functional representation of the brain. In fact, standard methodologies do not distinguish the contributions coming from different frequency bands, considering only one specific range. The resulting network provides a functional map of the brain and allows to identify special regions which act as hubs, i.e. units either with larger connectivity than others or with strategic functions which maximize the information flow through them [16, 17, 18, 19] (see Ref. [15] for a review). It is worth remarking that while the concept of information flow is well defined for structural networks, it requires to be careful in the case of functional ones. In fact, functional representations encode statistically significant correlations between brain regions and, strictly speaking, the concept of information flowing through such (often non-physical) links is

poorly defined. Here, the interpretation of some network descriptors in terms of information flow is given to better elucidate the meaning of the descriptor in a structural context, rather than to characterize physical information dynamics.

Given their functional importance, hubs mediate interactions among other regions and might favor the brain's integrated operation. They are generally identified by centrality descriptors [20] and they are of particular interest in many applications [21, 22, 23, 24]. Recent studies have shown that the importance of each region is subjected to dramatic changes depending on the frequency cuts [25] and that hubs might be very different when functional connectivity is measured in different frequency bands [26]. These results, together with previous findings concerning the importance of topological information measured from components above 0.1 Hz [27, 28, 29], suggest that a novel framework for modeling and analysis of human brain functional connectivity is required.

The new framework must be able to consider functional information from different frequency bands, simultaneously: in practice, for each band it is sufficient to build a functional network and then to analyze the resulting system as a whole. Multilayer networks provide the mathematical background [7] for this purpose. In this new framework, each region of the brain is mapped into a network node and replicated across all layers, encoding frequency bands, where they are connected with other nodes by means of functional links – corresponding to significant correlations in a specific frequency band. The methodology is summarized in the top panels of Fig. 2, while the result of the procedure applied to a real human brain is visualized in Fig. 3.

Nodes are interconnected with their replicas – also known as *state nodes* – across layers and the weight of these links is, in general, a free parameter which must be estimated from the data or by maximizing a specific cost function [30]. For each unit, the set of state nodes constitutes a *physical node* corresponding to a specific brain region. State nodes of a single physical node are interconnected categorically, i.e., they build a clique.

The first question to answer is to which extent such an enriched representation of functional connectivity is more valuable than other aggregated (or less rich) representations. The answer has been recently given in Ref. [30], where it has been shown that each functional layer – in a range between 0.01 Hz and 0.25 Hz, in steps of 0.02 Hz – provides unique information and should be neither aggregated with other layers nor neglected. The result is based on the analysis of structural reducibility [31], a modern technique grounded on information entropy.

The irreducibility of the multilayer functional representation of human brain raises the necessity for multilayer analysis of the underlying architecture and a few first results have been recently reported about the identification of hubs. In other contexts, it has been shown that hubs in a multilayer network might be dramatically different from hubs in each layer of the system [32]. An intuitive example is given in the following. Let us consider a two-layer system where a certain node is in the periphery of both networks, and let us consider that such a node is the only one in common to the two layers. It is clear that this node is crucial for the exchange of information between the two layers and, as a consequence, it will be most central with respect to this criterion. In a classical analysis, where the layers are considered separately, the node is still peripheral and it would be the less central[1].

The multilayer analysis of brain's regions centrality reveals that hubs are, in general, different from the hubs identified by standard methodologies based on single-layer network analysis. The most surprising finding is that such hubs can be used to distinguish, with high accuracy and sensitivity (above 80% in both cases), the brain of a schizophrenic patient from a healthy brain in resting state [30], thus improving our understanding of schizophrenia and opening the door to the analysis of other brain disorders within the same framework.

Magnetoencephalography (MEG) has been recently used in a similar spirit, with layers encoding the connectivity between neural oscillations within four frequency bands, namely alpha (8–13 Hz), beta (13–30 Hz), low gamma (30–50 Hz) and high gamma (50–100 Hz). In this context, the mean connection strength – averaged across the network where the functional connectivity between schizophrenic patients and controls differs most – has been used to gain new insights about within and between oscillatory frequencies [36]. Two regimes of multilayer network behavior have been identified in a system with five layers (bands 1–4 Hz, 4–8 Hz, 8–13 Hz, 13–30 Hz and 30–48 Hz): in the first regime layers are independent, while in the second regime they are highly dependent. Results suggest that healthy human brain operates at the transition point between these two regimes [37].

These studies provide evidence for and support the hypothesis that functional layers do not act as independent entities, suggesting the existence of mechanisms for integration and segregation of brain activity

[1]Here, information exchange can be modeled by bits diffusing through the system either along random walks [33] or shortest paths [34, 35] between two endpoints.

within and across different frequency bands. Very recently, a mechanistic model for this process has been proposed [38]. The authors have compared the performance of two models: in the model A, each brain region generates oscillations in a single frequency; in model B, each brain region can generate oscillations in multiple frequency bands. The model B, named multi-frequency model, does not take into account cross-frequency interactions but it still outperforms single-frequency model in reproducing empirical MEG data [38].

In a more recent work, MEG recordings during resting states in subjects affected by the Alzheimer's disease have been used to build a multilayer network where layers represent functional connectivity in different frequency bands (2–4 Hz, 4–8 Hz, 8–10.5 Hz, 10.5–13 Hz, 13–20 Hz, 20–30 Hz, 30–45 Hz). The study has provided evidence that regional connectivity in unhealthy subjects was abnormally distributed across frequency bands – a feature with no counterpart in healthy individuals – revealing an abnormal loss of inter-frequency centrality in memory-related association areas. The proposed methodology has led to high classification accuracy (78.4%) and sensitivity (91.1%) of subjects, confirming the superior performance of multilayer analysis as compared to more traditional approaches [39].

All the results briefly described in this section support and reinforce the possibility to adopt multilayer techniques as a potential non-invasive biomarkers for neurodegenerative diseases and mental disorders.

Time-varying network model and task-based decomposition

Instead of building functional layers in the frequency domain, it might be desirable to consider the brain activity in the time domain, because temporal changes and their mapping might be biologically meaningful. It is worth noting that historically this was, in fact, the first multilayer approach to the analysis of brain networks, even when a formal theory for this type of structure was not yet available [40].

Usually, the measured BOLD activity is divided into a series of time windows named snapshots, – which can be overlapping or not – and a pairwise measure of correlation between regions of interest is calculated to build a functional network for each snapshot. However, it is fundamental to remark that this processing phase is far from providing a rigorous and well established method to build functional networks from this type of data [41]. In practice, overlapping and non-overlapping windows are not statistically independent [42, 43], their length is a free parameter and their choice requires careful inspection of the data [44, 45] to avoid mapping spurious connectivity fluctuations.

The resulting network is a multilayer graph where each layer corresponds to a functional snapshot of brain activity. This approach has the advantage of building a static backbone of the underlying functional dynamic of human brain that can be used, for instance, to better understand how it operates during specific tasks or on the onset of an epileptic seizure. In this regard, multilayer networks describing how functional connectivity changes across time provide a richer framework than traditional approaches [46]. In this framework, state nodes are interconnected only with their subsequent replicas, like in a chain. This methodology, summarized in the bottom panels of Fig. 2, has opened the door to several studies and triggered the development of novel theoretical measures to identify the most influent brain regions during learning [47] and how they cluster together in functional modules [48], to cite some of them.

The multilayer model for time-varying networks can be used to explore the role of functional fluctuations while in resting state or performing specifying activities (see Ref. [3] for an up-to-date review), where in the latter case one defines a task-based representation of brain activity [48, 49]. This type of decomposition is of particular interest because it is possible to map the reconfiguration of brain regions' correlated activity between different tasks or during a learning process [50, 51].

Besides the variety of its applications, very recently, this novel framework has been used to better characterize high-level language processing in humans by using fMRI data from 22 human subjects, asked to perform a language comprehension task. While it is known that the activity of left frontal, temporal, and parietal cortices is very correlated – constituting a functional system – when an individual is performing a naturalistic language comprehension task or she is resting, it is still poorly understood how they become part of such an integrated functional system. By identifying functional modules within the multilayer framework, involving the generalization of classical modularity maximization to the multilayer domain [8], it has been shown that a stable core of mutually co-activating brain regions emerges mainly in the left hemisphere, whereas a periphery of brain regions is developed in the right hemisphere, while co-activating with different regions at different times. One might ask if it is required to use such a complicated computational tool for this purpose. While it is possible to perform community – or any other network descriptor – analysis in each layer separately, only by performing multilayer analysis it is possible to account for the continuity of communities – or centrality, influence, clusters, and so forth so on – over time, a key advantage that has no counterpart in other single-layer or aggregated approaches.

This result, heavily based on the multilayer analysis of functional brain connectivity, suggests the existence of trade-off between a region's specialization and its capacity for flexible network reconfiguration [52] and highlights the power of this novel analytical framework to improve our understanding of brain's functional dynamics.

While brain activity during a single task can be studied by means of a temporal network, it has been recently shown that the networks corresponding to different tasks can be used to encode the layers of a multitask multilayer topology [53]. At variance with the temporal networks described above, where replicated nodes are interconnected across layers following the arrow of time (i.e., node i in layer corresponding to snapshot τ is linked to node i in layer(s) corresponding to $\tau' > \tau$), interconnectivity in multitask networks is categorical (i.e., node i in layer α is linked to all of its replicas in layers $\beta \neq \alpha$). Results from this research direction indicate that several inter-region temporal patterns observed at rest are preserved during different tasks, suggesting the existence of a primary intrinsic functional network architecture – similar to the one observed in resting state – that is enriched by a secondary task-dependent functional connectivity [53].

Structural and functional decomposition

Understanding the interplay between brain structure, function and dynamics is a longstanding challenge [54, 55, 56, 57, 58, 2]. The novel multilayer framework provides a unique opportunity to study, simultaneously, structural and functional information and, in fact, it has been recently used for this purpose [59, 60].

The first study concerns motifs, specific subgraphs of reduced size (generally 3 or 4 nodes) that play a fundamental role for the stability of the underlying system and several functions [61]. The significance of a motif is usually estimated by its occurrence with respect to a null model of the network. While the relationship between structural and functional brain motifs has been studied in the past [62], in Ref. [59] the authors have exploited the recent mathematical advances in network analysis to investigate multiplex motifs [63].

In their setup, each multiplex network consists of two layers: one reflecting anatomical connectivity – inferred from Diffusion Magnetic Resonance Imaging – and one encoding functional relationships – inferred from functional Magnetic Resonance Imaging – among the brain regions of healthy subjects. In this context, multiplex motifs are potentially more informative than their single-layer (either structural or functional) counterparts taken separately, because a larger number of configurations, accounting for both layers simultaneously, is considered. The results indicate that when a physical connection between different brain regions coexists with a non-trivial positive correlation in their activities, the corresponding motif is statistically significant, i.e. it occurs more frequently than random expectation. As a consequence, this works provides further quantitative support to the hypothesis that functional connectivity is non-trivially constrained by brain architecture.

In the same spirit, another study explored the relationship between structure and function the Macaque cortical network [60]. In this case, the functional layer has been derived from simulated neural activity, whereas structural information is provided by anatomical connectivity. From the study of multiplex clustering, involving triangles of nodes on the two layers, the authors have investigated the emergence of functional connections that have no structural counterpart and the dependence of the multiplex network on the neural dynamical regime.

Conclusion and Outlook

Increasing evidences show that our understanding of human brain cannot prescind from using more complex multi-scale and multilayer models than a decade ago. The new models have to account for the hierarchical organization of the brain in both spatial and temporal dimensions, as well as its functional organization changes across temporal and frequency domains, while interplaying with the underlying structure. The recent advances in network science led to the development of a powerful mathematical framework for multilayer networks [7], topologies able to account for the simultaneous existence of different types of relationships between system's units and their variation over time [5, 6, 11].

The present epoch is mature enough for multilayer analysis of human brain, to investigate the functional role of brain regions in different domains. While the field is still in its infancy, intense research activity is ongoing, based on advanced mathematical models to represent structural and functional connectivity, their evolution over time and their interdependence. Network science is just coming out of the multilayer revolution, which triggered hundreds of applications in all disciplines, from life sciences to humanities, in a few years. While there are still many theoretical challenges to tackle, such as the definition of appropriate null models to compare against the connectivity of empirical multilayer systems [64, 65, 66, 67], the outcome of such a revolution already provides several computational tools to identify key units in multilayer systems [68, 7, 34, 35, 69, 32], determine their organization in modules [8, 9, 70, 71], reduce connectivity into simpler architectures [31] and discover the hierarchical organization of layers [72].

The application of some of these tools to the analysis of human brain provided exciting novel insights about its structure and function. Nevertheless, from a methodological point of view it is still a challenge to define a physical meaning for inter-layer connectivity, beyond purely mathematical or computational arguments. For instance, in multiplex networks representing multimodal connectivity or structural-functional relationships, as well as in time-varying networks, nodes are replicated across different layers are linked with each other: in the former all the replicas are interconnected, whereas in the latter only replicas corresponding to subsequent temporal snapshots are connected in order to preserve the underlying causal structure of the data. However, the weight to assign to these inter-layer connections is a free parameter, as in the case of frequency-based decompositions (in this last case, a partial solution might be given by the analysis of cross-frequency correlations).

In the next future, we expect more complex structural and dynamical models able to account for several types of information, simultaneously. Such models will incorporate multivariate information from different domains, e.g., space, time and frequency, and across different scales, from cellular level to entire brain regions, with the ultimate goal to shed light on how the interplay between structure and dynamics is related to brain diseases and give rise to cognition.

Competing interests
The author declares that he has no competing interests.

Author's contributions
The author conceived the idea for this correspondence, conceptualized and wrote this article.

Acknowledgements
The author acknowledges financial support from the Spanish program Juan de la Cierva (IJCI-2014-20225).

References
1. Bullmore, E., Sporns, O.: Complex brain networks: graph theoretical analysis of structural and functional systems. Nature Reviews Neuroscience **10**(3), 186–198 (2009)
2. Park, H.-J., Friston, K.: Structural and functional brain networks: from connections to cognition. Science **342**(6158), 1238411 (2013)
3. Betzel, R.F., Bassett, D.S.: Multi-scale brain networks. To appear in NeuroImage (arXiv:1608.08828) (2016)
4. Muldoon, S.F., Bassett, D.S.: Network and multilayer network approaches to understanding human brain dynamics. Philosophy of Science **83**(5), 710–720 (2016)
5. Kivelä, M., Arenas, A., Barthelemy, M., Gleeson, J.P., Moreno, Y., Porter, M.A.: Multilayer networks. Journal of complex networks **2**(3), 203–271 (2014)
6. Boccaletti, S., Bianconi, G., Criado, R., Del Genio, C.I., Gómez-Gardeñes, J., Romance, M., Sendiña-Nadal, I., Wang, Z., Zanin, M.: The structure and dynamics of multilayer networks. Physics Reports **544**(1), 1–122 (2014)
7. De Domenico, M., Solé-Ribalta, A., Cozzo, E., Kivelä, M., Moreno, Y., Porter, M.A., Gómez, S., Arenas, A.: Mathematical formulation of multilayer networks. Physical Review X **3**(4), 041022 (2013)
8. Mucha, P.J., Richardson, T., Macon, K., Porter, M.A., Onnela, J.-P.: Community structure in time-dependent, multiscale, and multiplex networks. science **328**(5980), 876–878 (2010)
9. De Domenico, M., Lancichinetti, A., Arenas, A., Rosvall, M.: Identifying modular flows on multilayer networks reveals highly overlapping organization in interconnected systems. Physical Review X **5**(1), 011027 (2015)
10. De Domenico, M., Solé-Ribalta, A., Omodei, E., Gómez, S., Arenas, A.: Ranking in interconnected multilayer networks reveals versatile nodes. Nature communications **6**, 6868 (2015)
11. De Domenico, M., Granell, C., Porter, M.A., Arenas, A.: The physics of spreading processes in multilayer networks. Nature Physics **12**, 901 (2016)
12. Cordes, D., Haughton, V.M., Arfanakis, K., Carew, J.D., Turski, P.A., Moritz, C.H., Quigley, M.A., Meyerand, M.E.: Frequencies contributing to functional connectivity in the cerebral cortex in ?resting-state? data. American Journal of Neuroradiology **22**(7), 1326–1333 (2001)
13. Cordes, D., Haughton, V., Carew, J.D., Arfanakis, K., Maravilla, K.: Hierarchical clustering to measure connectivity in fmri resting-state data. Magnetic resonance imaging **20**(4), 305–317 (2002)
14. Fox, M.D., Raichle, M.E.: Spontaneous fluctuations in brain activity observed with functional magnetic resonance imaging. Nature Reviews Neuroscience **8**(9), 700–711 (2007)
15. Fallani, F.D.V., Richiardi, J., Chavez, M., Achard, S.: Graph analysis of functional brain networks: practical issues in translational neuroscience. Phil. Trans. R. Soc. B **369**(1653), 20130521 (2014)
16. Van Den Heuvel, M.P., Pol, H.E.H.: Exploring the brain network: a review on resting-state fmri functional connectivity. European Neuropsychopharmacology **20**(8), 519–534 (2010)
17. Poldrack, R.A., Farah, M.J.: Progress and challenges in probing the human brain. Nature **526**(7573), 371–379 (2015)
18. Achard, S., Salvador, R., Whitcher, B., Suckling, J., Bullmore, E.: A resilient, low-frequency, small-world human brain functional network with highly connected association cortical hubs. The Journal of Neuroscience **26**(1), 63–72 (2006)
19. Power, J.D., Schlaggar, B.L., Lessov-Schlaggar, C.N., Petersen, S.E.: Evidence for hubs in human functional brain networks. Neuron **79**(4), 798–813 (2013)
20. Boccaletti, S., Latora, V., Moreno, Y., Chavez, M., Hwang, D.-U.: Complex networks: Structure and dynamics. Physics reports **424**(4), 175–308 (2006)
21. Sporns, O., Honey, C.J., Kötter, R.: Identification and classification of hubs in brain networks. PloS one **2**(10), 1049–1049 (2007)
22. Lynall, M.-E., Bassett, D.S., Kerwin, R., McKenna, P.J., Kitzbichler, M., Muller, U., Bullmore, E.: Functional connectivity and brain networks in schizophrenia. The Journal of Neuroscience **30**(28), 9477–9487 (2010)
23. Rubinov, M., Sporns, O.: Complex network measures of brain connectivity: uses and interpretations. Neuroimage **52**(3), 1059–1069 (2010)
24. Zuo, X.-N., Ehmke, R., Mennes, M., Imperati, D., Castellanos, F.X., Sporns, O., Milham, M.P.: Network centrality in the human functional connectome. Cerebral cortex **22**(8), 1862–1875 (2012)
25. Thompson, W.H., Fransson, P.: The frequency dimension of fmri dynamic connectivity: Network connectivity, functional hubs and integration in the resting brain. NeuroImage **121**, 227–242 (2015)
26. Sasai, S., Homae, F., Watanabe, H., Sasaki, A., Tanabe, H., Sadato, N., Taga, G.: Frequency-specific network topologies in the resting human brain. Frontiers in human neuroscience **8**, 1022 (2014)
27. Bassett, D.S., Meyer-Lindenberg, A., Achard, S., Duke, T., Bullmore, E.: Adaptive reconfiguration of fractal small-world human brain functional networks. PNAS **103**(51), 19518–19523 (2006)
28. Supekar, K., Menon, V., Rubin, D., Musen, M., Greicius, M.D., *et al.*: Network analysis of intrinsic functional brain connectivity in alzheimer?s disease. PLoS Comput Biol **4**(6), 1000100 (2008)
29. Chavez, M., Valencia, M., Navarro, V., Latora, V., Martinerie, J.: Functional modularity of background activities in normal and epileptic brain networks. Physical Review Letters **104**(11), 118701 (2010)
30. De Domenico, M., Sasai, S., Arenas, A.: Mapping multiplex hubs in human functional brain networks. Frontiers in Neuroscience **10**, 326 (2016)
31. De Domenico, M., Nicosia, V., Arenas, A., Latora, V.: Structural reducibility of multilayer networks. Nature communications **6**, 6864 (2015)

32. De Domenico, M., Solé-Ribalta, A., Omodei, E., Gómez, S., Arenas, A.: Ranking in interconnected multilayer networks reveals versatile nodes. Nature Communications **6**, 6868–6868 (2015)
33. De Domenico, M., Solé-Ribalta, A., Gómez, S., Arenas, A.: Navigability of interconnected networks under random failures. Proceedings of the National Academy of Sciences **111**(23), 8351–8356 (2014)
34. Battiston, F., Nicosia, V., Latora, V.: Structural measures for multiplex networks. Physical Review E **89**(3), 032804 (2014)
35. Solé-Ribalta, A., De Domenico, M., Gómez, S., Arenas, A.: Centrality rankings in multiplex networks. In: Proceedings of the 2014 ACM Conference on Web Science, pp. 149–155 (2014). ACM
36. Brookes, M.J., Tewarie, P.K., Hunt, B.A., Robson, S.E., Gascoyne, L.E., Liddle, E.B., Liddle, P.F., Morris, P.G.: A multi-layer network approach to meg connectivity analysis. NeuroImage **132**, 425–438 (2016)
37. Tewarie, P., Hillebrand, A., van Dijk, B.W., Stam, C.J., O'Neill, G.C., Van Mieghem, P., Meier, J.M., Woolrich, M.W., Morris, P.G., Brookes, M.J.: Integrating cross-frequency and within band functional networks in resting-state meg: A multi-layer network approach. NeuroImage **142**, 324–336 (2016)
38. Deco, G., Cabral, J., Woolrich, M., Stevner, A., van Hartevelt, T., Kringelbach, M.: Single or multi-frequency generators in on-going brain activity: a mechanistic whole-brain model of empirical meg data. bioRxiv:084103 (2016). doi:10.1101/084103. http://biorxiv.org/content/early/2016/11/04/084103.full.pdf
39. Guillon, J., Attal, Y., Colliot, O., La Corte, V., Dubois, B., Schwartz, D., Chavez, M., Fallani, F.D.V.: Loss of inter-frequency brain hubs in alzheimer's disease. arXiv:1701.00096 (2017)
40. Honey, C.J., Kötter, R., Breakspear, M., Sporns, O.: Network structure of cerebral cortex shapes functional connectivity on multiple time scales. Proceedings of the National Academy of Sciences **104**(24), 10240–10245 (2007)
41. Zalesky, A., Fornito, A., Cocchi, L., Gollo, L.L., Breakspear, M.: Time-resolved resting-state brain networks. Proceedings of the National Academy of Sciences **111**(28), 10341–10346 (2014)
42. Thompson, W.H., Fransson, P.: The mean–variance relationship reveals two possible strategies for dynamic brain connectivity analysis in fmri. Frontiers in human neuroscience **9** (2015)
43. Betzel, R.F., Fukushima, M., He, Y., Zuo, X.-N., Sporns, O.: Dynamic fluctuations coincide with periods of high and low modularity in resting-state functional brain networks. NeuroImage **127**, 287–297 (2016)
44. Leonardi, N., Van De Ville, D.: On spurious and real fluctuations of dynamic functional connectivity during rest. Neuroimage **104**, 430–436 (2015)
45. Zalesky, A., Breakspear, M.: Towards a statistical test for functional connectivity dynamics. Neuroimage **114**, 466–470 (2015)
46. Holme, P., Saramäki, J.: Temporal networks. Physics reports **519**(3), 97–125 (2012)
47. Mantzaris, A.V., Bassett, D.S., Wymbs, N.F., Estrada, E., Porter, M.A., Mucha, P.J., Grafton, S.T., Higham, D.J.: Dynamic network centrality summarizes learning in the human brain. Journal of Complex Networks **1**(1), 83–92 (2013)
48. Bassett, D.S., Wymbs, N.F., Porter, M.A., Mucha, P.J., Carlson, J.M., Grafton, S.T.: Dynamic reconfiguration of human brain networks during learning. PNAS **108**(18), 7641–7646 (2011)
49. Bassett, D.S., Wymbs, N.F., Rombach, M.P., Porter, M.A., Mucha, P.J., Grafton, S.T.: Task-based core-periphery organization of human brain dynamics. PLoS Comput Biol **9**(9), 1003171 (2013)
50. Mattar, M.G., Cole, M.W., Thompson-Schill, S.L., Bassett, D.S.: A functional cartography of cognitive systems. PLoS Comput Biol **11**(12), 1004533 (2015)
51. Bassett, D.S., Yang, M., Wymbs, N.F., Grafton, S.T.: Learning-induced autonomy of sensorimotor systems. Nature Neuroscience **18**(5), 744–751 (2015)
52. Chai, L.R., Mattar, M.G., Blank, I.A., Fedorenko, E., Bassett, D.S.: Functional network dynamics of the language system. Cerebral Cortex **26**, 4148–4159 (2016)
53. Cole, M.W., Bassett, D.S., Power, J.D., Braver, T.S., Petersen, S.E.: Intrinsic and task-evoked network architectures of the human brain. Neuron **83**(1), 238–251 (2014)
54. Cohen, J.D., Perlstein, W.M., Braver, T.S., Nystrom, L.E., Noll, D.C., Jonides, J., Smith, E.E., *et al.*: Temporal dynamics of brain activation during a working memory task. Nature **386**(6625), 604–608 (1997)
55. Sporns, O., Chialvo, D.R., Kaiser, M., Hilgetag, C.C.: Organization, development and function of complex brain networks. Trends in cognitive sciences **8**(9), 418–425 (2004)
56. Draganski, B., Gaser, C., Kempermann, G., Kuhn, H.G., Winkler, J., Büchel, C., May, A.: Temporal and spatial dynamics of brain structure changes during extensive learning. The Journal of Neuroscience **26**(23), 6314–6317 (2006)
57. Rubinov, M., Sporns, O., van Leeuwen, C., Breakspear, M.: Symbiotic relationship between brain structure and dynamics. BMC neuroscience **10**(1), 1 (2009)
58. Deisseroth, K., Schnitzer, M.J.: Engineering approaches to illuminating brain structure and dynamics. Neuron **80**(3), 568–577 (2013)
59. Battiston, F., Nicosia, V., Chavez, M., Latora, V.: Multilayer motif analysis of brain networks. To appear in Chaos (arXiv:1606.09115) (2016)
60. Crofts, J. J., Forrester, M., O'Dea, R. D.: Structure-function clustering in multiplex brain networks. EuroPhysics Letters **116**(1), 18003 (2016)
61. Milo, R., Shen-Orr, S., Itzkovitz, S., Kashtan, N., Chklovskii, D., Alon, U.: Network motifs: simple building blocks of complex networks. Science **298**(5594), 824–827 (2002)
62. Sporns, O., Kötter, R.: Motifs in brain networks. PLoS Biol **2**(11), 369 (2004)
63. Wernicke, S., Rasche, F.: Fanmod: a tool for fast network motif detection. Bioinformatics **22**(9), 1152–1153 (2006)
64. Bianconi, G., Dorogovtsev, S.N.: Multiple percolation transitions in a configuration model of a network of networks. Physical Review E **89**(6), 062814 (2014)
65. Cozzo, E., Kivelä, M., De Domenico, M., Solé-Ribalta, A., Arenas, A., Gómez, S., Porter, M.A., Moreno, Y.: Structure of triadic relations in multiplex networks. New Journal of Physics **17**(7), 073029 (2015)
66. Sarzynska, M., Leicht, E.A., Chowell, G., Porter, M.A.: Null models for community detection in spatially embedded, temporal networks. Journal of Complex Networks, 027 (2015)
67. Bazzi, M., Jeub, L.G., Arenas, A., Howison, S.D., Porter, M.A.: Generative benchmark models for mesoscale structure in multilayer networks. arXiv:1608.06196 (2016)
68. Tang, J., Musolesi, M., Mascolo, C., Latora, V., Nicosia, V.: Analysing information flows and key mediators through temporal centrality metrics. In: Proceedings of the 3rd Workshop on Social Network Systems, p. 3 (2010). ACM
69. Menichetti, G., Remondini, D., Panzarasa, P., Mondragón, R.J., Bianconi, G.: Weighted multiplex networks. PloS one **9**(6), 97857 (2014)
70. Peixoto, T.P.: Inferring the mesoscale structure of layered, edge-valued, and time-varying networks. Physical Review E **92**(4), 042807 (2015)
71. Valles-Catala, T., Massucci, F.A., Guimera, R., Sales-Pardo, M.: Multilayer stochastic block models reveal the multilayer structure of complex networks. Physical Review X **6**(1), 011036 (2016)
72. De Domenico, M., Biamonte, J.: Spectral entropies as information-theoretic tools for complex network comparison. Physical Review X **6**(4), 041062 (2016)
73. De Domenico, M., Porter, M.A., Arenas, A.: Muxviz: a tool for multilayer analysis and visualization of networks. Journal of Complex Networks **3**, 159–176 (2015)

Figures

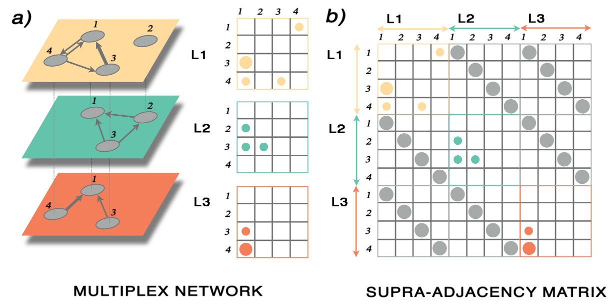

Figure 1 Multilayer network representation. (a) A multilayer network consists of different networks encoded by layers, each one represented by a (possibly directed and weighted) adjacency matrix. **(b)** The rank-4 multilayer adjacency tensor, representing intra- and inter-layer connectivity is generally flattened by matricization to a rank-2 tensor, generally known as supra-adjacency matrix, without loss of information.

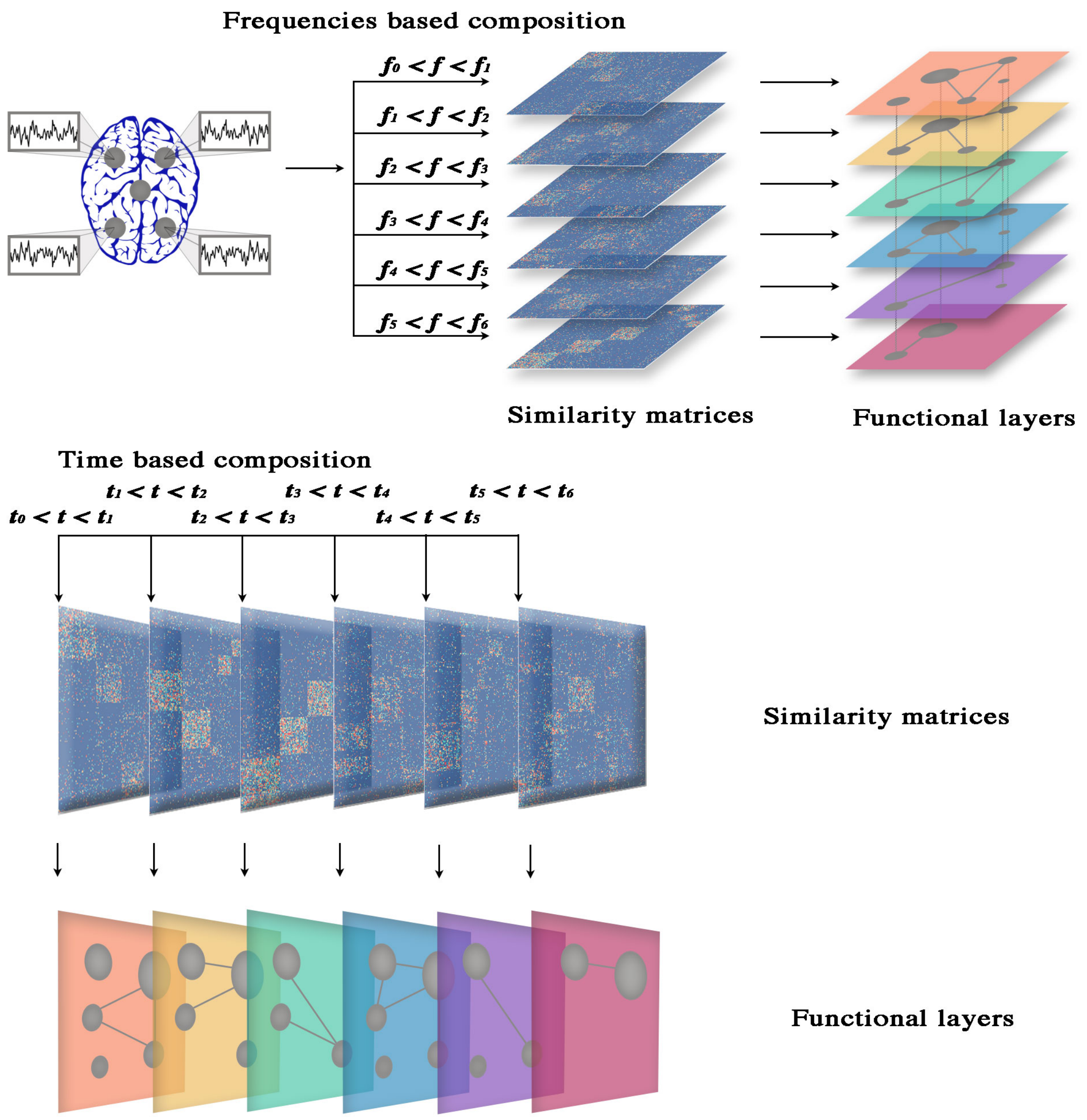

Figure 2 Building the multilayer functional brain. Top panels: brain activity is measured in different regions and signals are decomposed in the frequency domain. The frequency domain consists of (possibly overlapping) frequency bands and, for each band, coherence – or other similarity descriptors – is measured between all pairs of regions. A similarity matrix is built for each frequency domain and statistical analysis of significance is used to map each matrix into a functional network, constituting a functional layer of the overall multilayer system. **Bottom panels:** in this case, signals are decomposed in the time domain, which consists of (possibly overlapping) consecutive temporal snapshots. A similarity matrix is calculated for each snapshot and the corresponding functional layer is built.

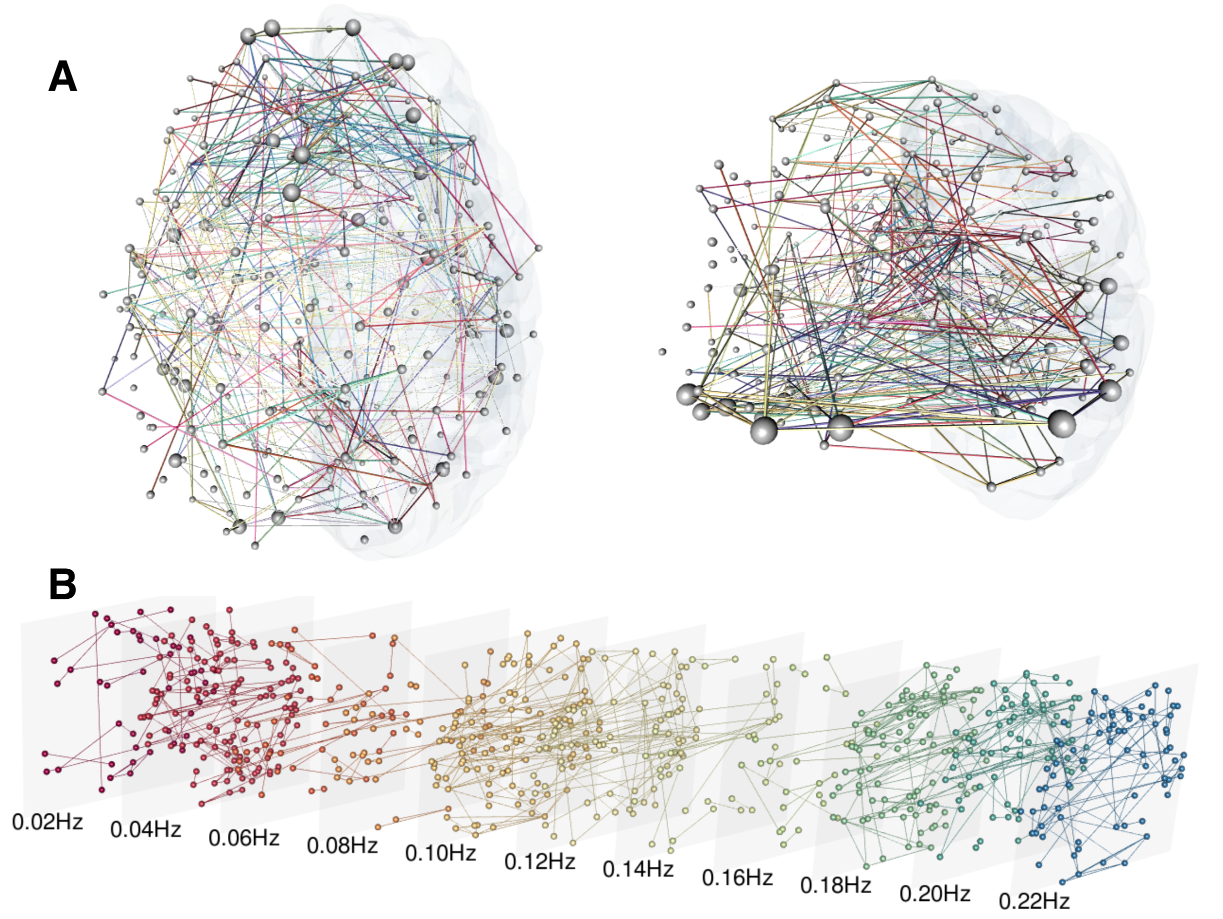

Figure 3 Visualizing the multilayer functional brain. Three-dimensional representations of the multilayer functional brain of a schizophrenic subject, based on frequency decomposition (11 layers, non-overlapping frequency bands between 0.01 Hz and 0.23 Hz). Only links with at least 6 standard deviations from the mean are shown (see Ref. [30] for further details). **Top panels:** edge-colored representation, where connections are colored according to the frequency band and node size is proportional to their functional versatility [30]. **Bottom panel:** multi-slice representation, where each layer encodes information about a specific frequency band [73] and inter-layer connectivity is not shown explicitly for sake of simplicity. The color scheme is the same in the two representations.

Dear Editor,

Thank you for editing our manuscript. We are glad that all the reviewers agree on the quality of the manuscript, its readability and its suitability for publication in *Gigascience*.

All the referees provided valuable suggestions that improved the quality of this review. We have taken into account all the recommendations, and a more detailed reply to each point is attached at the end of this letter.

Summarizing the changes:

- Extended Background section, to better explain structural and functional brain networks;
- Extended second section, to provide more details about salient features of different types of multilayer networks, with special focus to the ones of interest for neuroscience applications;
- Extended the section on frequency decomposition, to cover one study appeared while the manuscript was under review;
- Extended the time-varying network section, to include a more appropriate review of task-based decomposition;
- Modified the Conclusion section and extensively extended it to include outlooks.
- Added Figure 3, upon suggestion of several colleagues. The figure intends to show a practical visualization of an empirical multilayer functional brain from different perspectives.

All changes are highlighted with red font in the revised version of the manuscript, to facilitate their identification.

Note that we would like to remove the part “The rise of” from the title, because in our opinion the new title sounds more authoritative.

Sincerely,

Manlio De Domenico

Reviewer #1

This review deals with the important topic of multi-layer network methods for neuroscience. It is well-written, in general, and discusses many of the important papers sitting at the confluence of these two fields.

Thank you for your positive assessment of our manuscript.

I have little to add in terms of writing style and content, though I have a few suggestions for how the scope could be broadened and some of the discussions made to be more balanced. Throughout the review, the author discusses functional brain networks in terms of information transfer and transmission. I think that it is important to note that functional brain networks do not transfer "information" nor is "information" capable of flowing over their links or between layers (functional networks represent brainwide correlation patterns that arise as a consequence of some dynamics constrained by an underlying physical network (i.e. a structural or anatomical brain network). This is a point that is often overlooked, partly because the measures used to diagnose or infer information transfer are generally agnostic as to whether they are applied to a functional or structural network, e.g. one can compute betweenness centrality measures on both classes of networks, but the concept of shortest path structure in a network whose links are based on correlations or coherence estimates is vague. In short, I would suggest revising some of the statements on information transfer and functional networks (e.g. on p.2 when discussing information flow over multi-frequency networks).

We agree with the referee on this point and we have added the following text to avoid confusion in the reader:

It is worth remarking that while the concept of information flow is well defined for structural networks, it requires to be careful in the case of functional ones. In fact, functional representations encode statistically significant correlations between brain regions and, strictly speaking, the concept of information flowing through such (often non-physical) links is poorly defined. Here, the interpretation of some network descriptors in terms of information flow is given to better elucidate the meaning of the descriptor in a structural context, rather than to characterize physical information dynamics.

Another point that, in my opinion, should be noted, is the lack of rigorous methods for estimating time-varying functional connectivity. The author presents this topic as though constructing such networks is as simple as specifying a window length, calculating a correlation, and then aggregating the "snapshots." In general, the problem is more difficult -- for overlapping (and even non-overlapping) windows successive windows are not independent of one another [3, 1], the length of the window is a free parameter and needs to be chosen carefully [4, 2], whether to taper the window is an open question [5], and so on. The authors should at least touch on this idea and note that while the multi-layer framework may be prepared to deal with time-varying networks, the process of estimating the connectivity over time is not yet resolved.

We thank the referee for this valuable comment. We completely agree with the referee and, in the time-varying network section (where overlapping windows are mentioned for the first time), we have added a brief discussion about this point and we have cited the suggested references.

It is also the case that the author focuses primarily on networks estimated from MRI data. While it is true that most network-based analysis in the neurosciences is relegated to these

kinds of data, it would maximize the readibility and relevance of the article if the author could also include some discussion on how these approaches could influence, say, cellular connectomics (this is where the author could speculate a bit).

We agree with the referee, but or choice has been mainly driven by the fact that this review will be part of a thematic series on (f)MRI. Nevertheless, we have partially accounted for this comment in the Conclusion and Outlook section.

Finally, it would be good to touch on null models. The use of rewiring models for "static" networks is well-documented, but te appropriate models for time-varying or multi-frequency multi-layer networks are not as well understood. It would be nice to offer some discussion of the matter.

Thank you for pointing this out. It is worth remarking that the choice of null models for multilayer networks is still the subject of ongoing research. We have added a sentence, in the conclusions, where we mention this issue and we cite a few key papers touching it.

1. (p. 1) The author asserts that "[T]he most promising approach is to use multilayer networks". I would regard multilayer network analysis as an important and promising approach, but to say that it is the "most promising" is a bit of an overstatement and suggest deleting the word "most".

Fixed accordingly.

2. (p. 2) "The tensorial representation of multilayer networks allows to develop". Should this read "allows us to develop?"

Fixed.

3. (p. 2) "The majority of such tools is based on the analysis of how information spreads through the multilayer system (see Ref. [10] and references therein) and provides a suitable framework for the analysis of human brain". Information spreading makes sense when links in the networks being study represent physical connections (e.g. fiber pathways) along which signals could possibly spread. For functional networks, where connections represent statistical relationships (e.g. correlations) that arise as a consequence of some dynamics constrained by structural networks, it is less clear to me that the spreading model is appropriate. It might be nice for the author to comment on this.

The text added in response to one of the previous major comments should be valid also in this case. We prefer to not change the text (or add new text) where that sentence is present. Nevertheless, to avoid confusion in the reader, we specified the word “structural analysis”, to differentiate from the case of functional analysis.

4. (p. 2) The statement "From the perspective of a single unit, generally named physical node, inter-layer connectivity between the corresponding state nodes constitutes a clique" is a bit vacuous to me; it does not seem to follow from the previous sentence. Could the author contextualize this statement a bit better?

We have rephrased as following:

For each unit, the set of state nodes constitutes a physical node corresponding to a specific brain region. State nodes of a single physical node are interconnected categorically, i.e., they build a

clique.

5. (p. 2) Remove the comma in "The result, is based . . . ".

Fixed.

I hope that the authors find these comments useful. -Richard Betzel

We are grateful for the very useful comments.

Reviewer #2

This review highlights an important and emerging topic in network neuroscience: modeling human brain networks using a multilayer network framework. Multilayer network analysis is well suited for neuroscience data as described in the manuscript, but its use is still in its infancy, and the therefore this review will help to advance its use in the community. In general, the paper is well organized and covers the relevant material, however, there are some points that could use more clarification for unfamiliar readers and I have included some additional and useful references to incorporate.

We thank the referee for her/his positive assessment of our work.

1. Structural vs. functional networks. I think that not all readers will be automatically comfortable with the distinction between structural (representing anatomical connections) and functional (representing statistical relationships between nodal dynamics) networks. In the introduction it would be useful to more directly define and clarify this distinction.

2. Describing structural networks. The construction of functional networks is described throughout the paper, but how structural networks are defined is given less description. While the emphasis of the review is on constructing multilayer networks from these types of data, I think it is still useful to more clearly define what the structural networks represent as some readers might not know what information about anatomical structure can be extracted from diffusion MRI.

We agree with the referee on both points, and we have extended the introduction accordingly. A clear description of what is intended for structural and functional connectivity is provided, as well as a brief description of DTI, useful for the general reader.

3. Time-varying models and task-based decomposition. This section needs some clarification. This section only discusses temporal networks (with ordinal interlayer coupling), and I therefore find the section title misleading. (It is true that in one of the references, temporal networks are examined during learning of a motor task, but a temporal network is still what is being analyzed.) Perhaps I am just misinterpreting the section title, but I expected a discussion of categorical task networks where each layer represents activity during a different type of task (requiring an all-to-all interlayer coupling). Either this section should be renamed, or a discussion of a multitask network should be included (see Cole et al 2014).

Useful references:

Building multilayer networks from neuroscience data:

Muldoon, S. F., & Bassett, D. S. (2016). Network and Multilayer Network Approaches to Understanding Human Brain Dynamics. Philosophy of Science, 83(5), 710-720.

Catagorical Task Networks:
Cole, M. W., Bassett, D. S., Power, J. D., Braver, T. S., & Petersen, S. E. (2014). Intrinsic and Task-Evoked Network Architectures of the Human Brain. Neuron, 83(1), 238-251.

We thank the referee for this valuable comment. Accordingly, we have extended the corresponding section by adding a paragraph where the work by Cole et al is briefly summarized. The work by Muldoon and Bassett has been cited in the Background section.

Reviewer #3

This paper reviewed the evolution of multilayer modeling framework and its contributions on human brain network analysis. In this framework, brain regions are considered as connected nodes which form a complicated network of multiple layers. These layers could represent different structural regions, functional levels, and also temporal activities. In this paper, the author gave a very comprehensive review over the existing studies in multilayer modeling. Most of current research for this topic is discussed with suitable arguments. The paper is well organized and the figures are illustrative to show the ideas behind. I think this is a good paper that could earn an acceptance after some minor revisions. For example, I think it would be better if the authors could provide more comparisons over existing methods if the page space is still available. It would helpful for other researchers if the author could give a brief outlook about the multilayer modeling in analysis of human brain network. There are still some typos/grammar errors need to be corrected. This includes but is not limited to the following examples. In the last paragraph of Page 2, 'The result, is based on' could be 'The result is based on'. In the first paragraph of Page 3, the comma is over used. It will be better if the long sentence is partitioned into short ones.

We thank the reviewer for his/her very positive assessment of our manuscript.

Following the first suggestion, we have extended the Conclusion section (now Conclusion and Outlook) with the requested brief outlook.

We have fixed the typo(s) and partitioned the long sentence into two shorter ones.
