# Supplementary material for: Multilayer modeling and analysis of human brain networks
Source: Gigascience. 2017 Feb 6;6(5):1–8. doi: 10.1093/gigascience/gix004 (PMC5437946; doi:10.1093/gigascience/gix004)
Supplement: Reviewer_1_Original_Submission_(Attachement_).pdf [file gix004_Reviewer_1_Original_Submission_(Attachement_).pdf]

The rise of multilayer modeling and analysis of human brain networks

December 27, 2016

Comments

This review deals with the important topic of multi-layer network methods for neuroscience. It is well-written, in general, and discusses many of the important papers sitting at the confluence of these two fields. I have little to add in terms of writing style and content, though I have a few suggestions for how the scope could be broadened and some of the discussions made to be more balanced.

Throughout the review, the author discusses functional brain networks in terms of information transfer and transmission. I think that it is important to note that functional brain networks do not transfer "information" nor is "information" capable of flowing over their links or between layers – functional networks represent brainwide correlation patterns that arise as a consequence of some dynamics constrained by an underlying physical network (i.e. a structural or anatomical brain network).

This is a point that is often overlooked, partly because the measures used to diagnose or infer information transfer are generally agnostic as to whether they are applied to a functional or structural network, e.g. one can compute betweenness centrality measures on both classes of networks, but the concept of shortest path structure in a network whose links are based on correlations or coherence estimates is vague. In short, I would suggest revising some of the statements on information transfer and functional networks (e.g. on p. 2 when discussing information flow over multi-frequency networks).

Another point that, in my opinion, should be noted, is the lack of rigorous methods for estimating time-varying functional connectivity. The author presents this topic as though constructing such networks is as simple as specifying a window length, calculating a correlation, and then aggregating the"snapshots." In general, the problem is more difficult – for overlapping (and even non-overlapping) windows successive windows are not independent of one another [3, 1], the length of the window is a free parameter and needs to be chosen carefully [4, 2], whether to taper the window is an open question [5], and so on. The authors should at least touch on this idea and note that while the multi-layer framework may be prepared to deal with time-varying networks, the process of estimating the connectivity over time is not yet resolved.

It is also the case that the author focuses primarily on networks estimated from MRI data. While it is true that most network-based analysis in the neurosciences is relegated to these kinds of data, it would maximize the readibility and relevance of the article if the author could also include some discussion on how these approaches could influence, say, celluar connectomics (this is where the author could speculate a bit).

Finally, it would be good to touch on null models. The use of rewiring models for "static" networks is well-documented, but te appropriate models for time-varying or multi-frequency multi-layer networks are not as well understood. It would be nice to offer some discussion of the matter.

1. (p. 1) The author asserts that *"[T]he most promising approach is to use multilayer networks".* I would regard multilayer network analysis as an important and promising approach, but to say that it is the "most promising" is a bit of an overstatement and suggest deleting the word "most".

2. (p. 2) *"The tensorial representation of multilayer networks allows to develop".* Should this read "allows us to develop?"

3. (p. 2) *"The majority of such tools is based on the analysis of how information spreads through the multilayer system (see Ref. [10] and references therein) and provides a suitable framework for the analysis of human brain"*. Information spreading makes sense when links in the networks being study represent physical connections (e.g. fiber pathways) along which signals could possibly spread. For functional networks, where connections represent statistical relationships (e.g. correlations) that arise as a consequence of some dynamics constrained by structural networks, it is less clear to me that the spreading model is appropriate. It might be nice for the author to comment on this.

4. (p. 2) The statement *"From the perspective of a single unit, generally named physical node, inter-layer connectivity between the corresponding state nodes constitutes a clique"* is a bit vacuous to me; it does not seem to follow from the previous sentence. Could the author contextualize this statement a bit better?

5. (p. 2) Remove the comma in *"The result, is based . . . "*.

I hope that the authors find these comments useful. -Richard Betzel

References

[1] Richard F Betzel, Makoto Fukushima, Ye He, Xi-Nian Zuo, and Olaf Sporns. Dynamic fluctuations coincide with periods of high and low modularity in resting-state functional brain networks. *NeuroImage*, 127:287–297, 2016.

[2] Nora Leonardi and Dimitri Van De Ville. On spurious and real fluctuations of dynamic functional connectivity during rest. *Neuroimage*, 104:430–436, 2015.

[3] William H Thompson and Peter Fransson. The mean–variance relationship reveals two possible strategies for dynamic brain connectivity analysis in fmri. *Frontiers in human neuroscience*, 9, 2015.

[4] Andrew Zalesky and Michael Breakspear. Towards a statistical test for functional connectivity dynamics. *Neuroimage*, 114:466–470, 2015.

[5] Andrew Zalesky, Alex Fornito, Luca Cocchi, Leonardo L Gollo, and Michael Breakspear. Time-resolved resting-state brain networks. *Proceedings of the National Academy of Sciences*, 111(28):10341–10346, 2014.
