# Supplementary material for: Multilayer modeling and analysis of human brain networks
Source: Gigascience. 2017 Feb 6;6(5):1–8. doi: 10.1093/gigascience/gix004 (PMC5437946; doi:10.1093/gigascience/gix004)
Supplement: Response_to_Reviewer_Comments_Original_Submission.pdf [file gix004_Response_to_Reviewer_Comments_Original_Submission.pdf]

Dear Editor,

Thank you for editing our manuscript. We are glad that all the reviewers agree on the quality of the manuscript, its readability and its suitability for publication in Gigascience.

All the referees provided valuable suggestions that improved the quality of this review. We have taken into account all the recommendations, and a more detailed reply to each point is attached at the end of this letter.

Summarizing the changes:

- Extended Background section, to better explain structural and functional brain networks;
- Extended second section, to provide more details about salient features of different types of multilayer networks, with special focus to the ones of interest for neuroscience applications;
- Extended the section on frequency decomposition, to cover one study appeared while the manuscript was under review;
- Extended the time-varying network section, to include a more appropriate review of task-based decomposition;
- Modified the Conclusion section and extensively extended it to include outlooks.
- Added Figure 3, upon suggestion of several colleagues. The figure intends to show a practical visualization of an empirical multilayer functional brain from different perspectives.

All changes are highlighted with red font in the revised version of the manuscript, to facilitate their identification.

Note that we would like to remove the part "The rise of" from the title, because in our opinion the new title sounds more authoritative.

Sincerely,

Manlio De Domenico

Reviewer #1

* This review deals with the important topic of multi-layer network methods for neuroscience. It is well-written, in general, and discusses many of the important papers sitting at the confluence of these two

fields.

Thank you for your positive assessment of our manuscript.

* I have little to add in terms of writing style and content, though I have a few suggestions for how the scope could be broadened and some of the discussions made to be more balanced. Throughout the review, the author discusses functional brain networks in terms of information transfer and transmission. I think that it is important to note that functional brain networks do not transfer "information" nor is "information" capable of flowing over their links or between layers (functional networks represent brainwide correlation patterns that arise as a consequence of some dynamics constrained by an underlying physical network (i.e. a structural or anatomical brain network). This is a point that is often overlooked, partly because the measures used to diagnose or infer information transfer are generally agnostic as to whether they are applied to a functional or structural network, e.g. one can compute betweenness centrality measures on both classes of networks, but the concept of shortest path structure in a network whose links are based on correlations or coherence estimates is vague. In short, I would suggest revising some of the statements on information transfer and functional networks (e.g. on p.2 when discussing information flow over multi-frequency networks).

We agree with the referee on this point and we have added the following text to avoid confusion in the reader:

It is worth remarking that while the concept of information flow is well defined for structural networks, it requires to be careful in the case of functional ones. In fact, functional representations encode statistically significant correlations between brain regions and, strictly speaking, the concept of information flowing through such (often non-physical) links is poorly defined. Here, the interpretation of some network descriptors in terms of information flow is given to better elucidate the meaning of the descriptor in a structural context, rather than to characterize physical information dynamics.

* Another point that, in my opinion, should be noted, is the lack of rigorous methods for estimating time-varying functional connectivity. The author presents this topic as though constructing such networks is as simple as specifying a window length, calculating a correlation, and then aggregating the "snapshots." In general, the problem is more difficult -- for overlapping (and even non-overlapping) windows successive windows are not independent of one another [3, 1], the length of the window is a free parameter and needs to be chosen carefully [4, 2], whether to taper the window is an open question [5], and so on. The authors should at least touch on this idea and note that while the multi-layer framework may be prepared to deal with time-varying networks, the process of estimating the connectivity over time is not yet resolved.

We thank the referee for this valuable comment. We completely agree with the referee and, in the time-varying network section (where overlapping windows are mentioned for the first time), we have added a brief discussion about this point and we have cited the suggested references.

*It is also the case that the author focuses primarily on networks estimated from MRI data. While it is true that most network-based analysis in the neurosciences is relegated to these kinds of data, it would maximize the readibility and relevance of the article if the author could also include some discussion on how these approaches could influence, say, cellular connectomics (this is where the author could speculate a bit).

We agree with the referee, but or choice has been mainly driven by the fact that this review will be part of a thematic series on (f)MRI. Nevertheless, we have partially accounted for this comment in the Conclusion and Outlook section.

* Finally, it would be good to touch on null models. The use of rewiring models for "static" networks is well-documented, but te appropriate models for time-varying or multi-frequency multi-layer networks are not as well understood. It would be nice to offer some discussion of the matter.

Thank you for pointing this out. It is worth remarking that the choice of null models for multilayer networks is still the subject of ongoing research. We have added a sentence, in the conclusions, where we mention this issue and we cite a few key papers touching it.

* 1. (p. 1) The author asserts that "[T]he most promising approach is to use multilayer networks". I would regard multilayer network analysis as an important and promising approach, but to say that it is the "most promising" is a bit of an overstatement and suggest deleting the word "most".

Fixed accordingly.

* 2. (p. 2) "The tensorial representation of multilayer networks allows to develop". Should this read "allows us to develop?"

Fixed.

* 3. (p. 2) "The majority of such tools is based on the analysis of how information spreads through the multilayer system (see Ref. [10] and references therein) and provides a suitable framework for the analysis of human brain". Information spreading makes sense when links in the networks being study represent physical connections (e.g. fiber pathways) along which signals could possibly spread. For functional networks, where connections represent statistical relationships (e.g. correlations) that arise as a consequence of some dynamics constrained by structural networks, it is less clear to me that the spreading model is appropriate. It might be nice for the author to comment on this.

The text added in response to one of the previous major comments should be valid also in this case. We prefer to not change the text (or add new text) where that sentence is present. Nevertheless, to avoid confusion in the reader, we specified the word “structural analysis”, to differentiate from the case of functional analysis.

* 4. (p. 2) The statement "From the perspective of a single unit, generally named physical node, inter-layer connectivity between the corresponding state nodes constitutes a clique" is a bit vacuous to me; it does not seem to follow from the previous sentence. Could the author contextualize this statement a bit better?

We have rephrased as following:

For each unit, the set of state nodes constitutes a physical node corresponding to a specific brain region. State nodes of a single physical node are interconnected categorically, i.e., they build a clique.

* 5. (p. 2) Remove the comma in "The result, is based . . . ".

Fixed.

* I hope that the authors find these comments useful. -Richard Betzel

We are grateful for the very useful comments.

Reviewer #2

*This review highlights an important and emerging topic in network neuroscience: modeling human brain networks using a multilayer network framework. Multilayer network analysis is well suited for neuroscience data as described in the manuscript, but its use is still in its infancy, and the therefore this review will help to advance its use in the community. In general, the paper is well organized and covers the relevant material, however, there are some points that could use more clarification for unfamiliar readers and I have included some additional and useful references to incorporate.

We thank the referee for her/his positive assessment of our work.

* 1. Structural vs. functional networks. I think that not all readers will be automatically comfortable with the distinction between structural (representing anatomical connections) and functional (representing statistical relationships between nodal dynamics) networks. In the introduction it would be useful to more directly define and clarify this distinction.

2. Describing structural networks. The construction of functional networks is described throughout the paper, but how structural networks are defined is given less description. While the emphasis of the review is on constructing multilayer networks from these types of data, I think it is still useful to more clearly define what the structural networks represent as some readers might not know what information about anatomical structure can be extracted from diffusion MRI.

We agree with the referee on both points, and we have extended the introduction accordingly. A clear

description of what is intended for structural and functional connectivity is provided, as well as a brief description of DTI, useful for the general reader.

* 3. Time-varying models and task-based decomposition. This section needs some clarification. This section only discusses temporal networks (with ordinal interlayer coupling), and I therefore find the section title misleading. (It is true that in one of the references, temporal networks are examined during learning of a motor task, but a temporal network is still what is being analyzed.) Perhaps I am just misinterpreting the section title, but I expected a discussion of categorical task networks where each layer represents activity during a different type of task (requiring an all-to-all interlayer coupling). Either this section should be renamed, or a discussion of a multitask network should be included (see Cole et al 2014).

Useful references:

Building multilayer networks from neuroscience data:
Muldoon, S. F., & Bassett, D. S. (2016). Network and Multilayer Network Approaches to Understanding Human Brain Dynamics. Philosophy of Science, 83(5), 710-720.

Catagorical Task Networks:
Cole, M. W., Bassett, D. S., Power, J. D., Braver, T. S., & Petersen, S. E. (2014). Intrinsic and Task-Evoked Network Architectures of the Human Brain. Neuron, 83(1), 238-251.

We thank the referee for this valuable comment. Accordingly, we have extended the corresponding section by adding a paragraph where the work by Cole et al is briefly summarized. The work by Muldoon and Bassett has been cited in the Background section.

Reviewer #3

* This paper reviewed the evolution of multilayer modeling framework and its contributions on human brain network analysis. In this framework, brain regions are considered as connected nodes which form a complicated network of multiple layers. These layers could represent different structural regions, functional levels, and also temporal activities. In this paper, the author gave a very comprehensive review over the existing studies in multilayer modeling. Most of current research for this topic is discussed with suitable arguments. The paper is well organized and the figures are illustrative to show the ideas behind. I think this is a good paper that could earn an acceptance after some minor revisions. For example, I think it would be better if the authors could provide more comparisons over existing methods if the page space is still available. It would helpful for other researchers if the author could give a brief outlook about the multilayer modeling in analysis of human brain network. There are still some typos/grammar errors need to be corrected. This includes but is not limited to the following examples.

In the last paragraph of Page 2, 'The result, is based on' could be 'The result is based on'. In the first paragraph of Page 3, the comma is over used. It will be better if the long sentence is partitioned into short ones.

We thank the reviewer for his/her very positive assessment of our manuscript.

Following the first suggestion, we have extended the Conclusion section (now Conclusion and Outlook) with the requested brief outlook.

We have fixed the typo(s) and partitioned the long sentence into two shorter ones.
