# Supplementary material for: Multilayer modeling and analysis of human brain networks
Source: Gigascience. 2017 Feb 6;6(5):1–8. doi: 10.1093/gigascience/gix004 (PMC5437946; doi:10.1093/gigascience/gix004)
Supplement: Reviewer_2_Report_(Original_Submission).pdf [file gix004_Reviewer_2_Report_(Original_Submission).pdf]

Reviewer Report

Title: "Multilayer modeling and analysis of human brain networks"

Version: Original Submission **Date:** 1/12/2017

Reviewer name: Sarah Muldoon

Reviewer Comments to Author:

This review highlights an important and emerging topic in network neuroscience: modeling human brain networks using a multilayer network framework. Multilayer network analysis is well suited for neuroscience data as described in the manuscript, but its use is still in its infancy, and the therefore this review will help to advance its use in the community. In general, the paper is well organized and covers the relevant material, however, there are some points that could use more clarification for unfamiliar readers and I have included some additional and useful references to incorporate.

1. Structural vs. functional networks. I think that not all readers will be automatically comfortable with the distinction between structural (representing anatomical connections) and functional (representing statistical relationships between nodal dynamics) networks. In the introduction it would be useful to more directly define and clarify this distinction.

2. Describing structural networks. The construction of functional networks is described throughout the paper, but how structural networks are defined is given less description. While the emphasis of the review is on constructing multilayer networks from these types of data, I think it is still useful to more clearly define what the structural networks represent as some readers might not know what information about anatomical structure can be extracted from diffusion MRI.

3. Time-varying models and task-based decomposition. This section needs some clarification. This section only discusses temporal networks (with ordinal interlayer coupling), and I therefore find the section title misleading. (It is true that in one of the references, temporal networks are examined during learning of a motor task, but a temporal network is still what is being analyzed.) Perhaps I am just misinterpreting the section title, but I expected a discussion of categorical task networks where each layer represents activity during a different type of task (requiring an all-to-all interlayer coupling). Either this section should be renamed, or a discussion of a multitask network should be included (see Cole et al 2014).

Useful references:

Building multilayer networks from neuroscience data:

Muldoon, S. F., & Bassett, D. S. (2016). Network and Multilayer Network Approaches to Understanding Human Brain Dynamics. Philosophy of Science, 83(5), 710-720.

Catagorical Task Networks:

Cole, M. W., Bassett, D. S., Power, J. D., Braver, T. S., & Petersen, S. E. (2014). Intrinsic and Task-Evoked Network Architectures of the Human Brain. Neuron, 83(1), 238-251.

Level of Interest

Please indicate how interesting you found the manuscript: An article of importance in its field

Quality of Written English

Please indicate the quality of language in the manuscript: Acceptable

Declaration of Competing Interests

Please complete a declaration of competing interests, considering the following questions:

- Have you in the past five years received reimbursements, fees, funding, or salary from an organisation that may in any way gain or lose financially from the publication of this manuscript, either now or in the future?
- Do you hold any stocks or shares in an organisation that may in any way gain or lose financially from the publication of this manuscript, either now or in the future?
- Do you hold or are you currently applying for any patents relating to the content of the manuscript?
- Have you received reimbursements, fees, funding, or salary from an organization that holds or has applied for patents relating to the content of the manuscript?
- Do you have any other financial competing interests?
- Do you have any non-financial competing interests in relation to this paper?

If you can answer no to all of the above, write 'I declare that I have no competing interests' below. If your reply is yes to any, please give details below.

I declare that I have no competing interests

I agree to the open peer review policy of the journal. I understand that my name will be included on my report to the authors and, if the manuscript is accepted for publication, my named report including any attachments I upload will be posted on the website along with the authors' responses. I agree for my report to be made available under an Open Access Creative Commons CC-BY license (http://creativecommons.org/licenses/by/4.0/). I understand that any comments which I do not wish to be included in my named report can be included as confidential comments to the editors, which will not be published.

I agree to the open peer review policy of the journal
