# Supplementary material for: Multilayer modeling and analysis of human brain networks
Source: Gigascience. 2017 Feb 6;6(5):1–8. doi: 10.1093/gigascience/gix004 (PMC5437946; doi:10.1093/gigascience/gix004)
Supplement: Reviewer_3_Report_(Original_Submission).docx [file gix004_Reviewer_3_Report_(Original_Submission).docx]

**Reviewer Report**

**Title:** "Multilayer modeling and analysis of human brain networks"

**Version:** Original Submission	**Date:** 1/15/2017

**Reviewer name:** Rongjian Li

**Reviewer Comments to Author:**

This paper reviewed the evolution of multilayer modeling framework and its contributions on human brain network analysis. In this framework, brain regions are considered as connected nodes which form a complicated network of multiple layers. These layers could represent different structural regions, functional levels, and also temporal activities. In this paper, the author gave a very comprehensive review over the existing studies in multilayer modeling. Most of current research for this topic is discussed with suitable arguments. The paper is well organized and the figures are illustrative to show the ideas behind. I think this is a good paper that could earn an acceptance after some minor revisions. For example, I think it would be better if the authors could provide more comparisons over existing methods if the page space is still available. It would helpful for other researchers if the author could give a brief outlook about the multilayer modeling in analysis of human brain network. There are still some typos/grammar errors need to be corrected. This includes but is not limited to the following examples. In the last paragraph of Page 2, 'The result, is based on' could be 'The result is based on'. In the first paragraph of Page 3, the comma is over used. It will be better if the long sentence is partitioned into short ones.

**Level of Interest**

Please indicate how interesting you found the manuscript: An article of importance in its field

**Quality of Written English**

Please indicate the quality of language in the manuscript: Needs some language corrections before being published

**Declaration of Competing Interests**

Please complete a declaration of competing interests, considering the following questions:

- Have you in the past five years received reimbursements, fees, funding, or salary from an organisation that may in any way gain or lose financially from the publication of this manuscript, either now or in the future?
- Do you hold any stocks or shares in an organisation that may in any way gain or lose financially from the publication of this manuscript, either now or in the future?
- Do you hold or are you currently applying for any patents relating to the content of the manuscript?
- Have you received reimbursements, fees, funding, or salary from an organization that holds or has applied for patents relating to the content of the manuscript?
- Do you have any other financial competing interests?
- Do you have any non-financial competing interests in relation to this paper?

If you can answer no to all of the above, write 'I declare that I have no competing interests' below. If your reply is yes to any, please give details below.

I declare that I have no competing interests.

I agree to the open peer review policy of the journal. I understand that my name will be included on my report to the authors and, if the manuscript is accepted for publication, my named report including any attachments I upload will be posted on the website along with the authors' responses. I agree for my report to be made available under an Open Access Creative Commons CC-BY license (http://creativecommons.org/licenses/by/4.0/). I understand that any comments which I do not wish to be included in my named report can be included as confidential comments to the editors, which will not be published.

I agree to the open peer review policy of the journal
